# The prokaryotic activity of the IGR IRESs is mediated by ribosomal protein S1

**DOI:** 10.1093/nar/gkac697

**Published:** 2022-08-30

**Authors:** Luc Roberts, Hans-Joachim Wieden

**Affiliations:** Alberta RNA Research and Training Institute, Department of Chemistry and Biochemistry, University of Lethbridge, Lethbridge, Alberta, Canada; Alberta RNA Research and Training Institute, Department of Chemistry and Biochemistry, University of Lethbridge, Lethbridge, Alberta, Canada; Department of Microbiology, University of Manitoba, Winnipeg, Manitoba, Canada

## Abstract

Internal ribosome entry sites (IRESs) are RNA elements capable of initiating translation on an internal portion of a messenger RNA. The intergenic region (IGR) IRES of the Dicistroviridae virus family folds into a triple pseudoknot tertiary structure, allowing it to recruit the ribosome and initiate translation in a structure dependent manner. This IRES has also been reported to drive translation in *Escherichia coli* and to date is the only described translation initiation signal that functions across domains of life. Here we show that unlike in the eukaryotic context the tertiary structure of the IGR IRES is not required for prokaryotic ribosome recruitment. In *E. coli* IGR IRES translation efficiency is dependent on ribosomal protein S1 in conjunction with an AG-rich Shine-Dalgarno-like element, supporting a model where the translational activity of the IGR IRESs is due to S1-mediated canonical prokaryotic translation.

## INTRODUCTION

Translation is one of the most fundamental processes occurring in every living cell (1–3). Consistent with its central role, translation is structurally and functionally conserved across all domains of life and can be divided into four main phases: initiation, elongation, termination, and recycling (2). Canonical eukaryotic translation initiation relies on the presence of the 7-methylguanylate cap at the 5′ end of messenger RNA (mRNA), which is recognized by initiation factors, that recruit the ribosome to initiate translation of the encoded peptide from the capped 5′ end of the mRNA (3). While this is the predominant form of eukaryotic translation initiation, numerous non-canonical mechanisms exist (4,5). Among these the internal ribosome entry sites (IRESs) are RNA elements present in a respective mRNA and that are capable of recruiting ribosomes. Through IRESs, translation initiation can begin on an internal portion of the mRNA (6). While all IRESs function independently of the 5′ cap they often require additional protein factors for their function (2). The intergenic region (IGR) IRES of the Dicistroviridae virus family is unique, as it does not require any initiation factors and initiates translation on a non-AUG start codon (7–9). This non-canonical activity relies on a triple pseudoknot (PK) tertiary structure, which recruits the ribosomal subunits and mimics a canonical tRNA–mRNA duplex (10–17). The IGR IRESs are phylogenetically classified into three subtypes based on the number of stem loops and the length of the loop regions (6,18). Subtypes I and II are well studied and similar in structure with subtype II having an extra hairpin in PKI, while subtype III is more recently discovered and significantly smaller than the other two subtypes (2,6,19–21). Interestingly, and in tone with the overall evolutionary conservation of the translation machinery, it has been reported that the IGR IRES from the *Plautia stali* intestine virus is able to also initiate translation in *Escherichia coli* (22). However, the reported prokaryotic IRES activity differs from that in the eukaryotic context. In *E. coli* the AUG start codon is essential for IRES mediated translation, additionally disrupting the PK structures demonstrated to be essential for translation initiation in eukaryotes has minimal effects on translation efficiency in *E. coli* (22). As a consequence, the proposed mechanism for prokaryotic IRES activity is a hybrid of the previously described eukaryotic IRES activity and the canonical bacterial Shine-Dalgarno (SD) initiation mechanisms whereby bacterial ribosomes transiently interact (in a structure dependent manner) with the IRES before repositioning to a downstream SD-like sequence (22,23). This suggests some structured mRNAs can indeed be specifically translated in bacteria by exploiting the evolutionary conserved structural features of the ribosomal core. Besides being interesting from an evolutionary perspective this is also of great interest and utility for bioengineers and synthetic biologists alike (22,24). Interested in characterizing the molecular mechanism underpinning this phenomenon, we performed an in-depth characterization of prokaryotic IGR IRES ribosome recruitment (*in vitro*) and translation (*in vivo* and *in vitro*). Surprisingly, our results demonstrate that the tertiary structure of the IGR IRES is dispensable for prokaryotic ribosome recruitment and translation initiation in *E. coli*. Instead, we find that IGR IRES translation efficiency correlates positively with single stranded AG-rich regions, is dependent on ribosomal protein S1, and requires a SD-like sequence upstream of the start codon. This challenges the previously proposed hybrid model in support of the canonical model of prokaryotic translation initiation for these IRESs where the bacterial translation machinery is not inherently biased to translate specific structured mRNAs, but rather seems to allow for translation of any mRNA for which the structure can be resolved by ribosomal protein S1 and where a SD-like sequence upstream of a start codon can be identified.

## MATERIALS AND METHODS

### Fluorescent reporter construct design

IRES reporter constructs were designed to adhere to BioBrick engineering standards (25). BioBrick Prefix and Suffix sequences (RFC 10) flank each construct for ease of cloning into BioBrick vectors with standardized copy numbers. A T7 promoter (BBa_I719005) drives the transcription of optimized superfolder green fluorescent protein (sfGFP) coding sequence translationally controlled by an RBS or IRES sequences. Transcription is stopped by a transcriptional terminator (BBa_B0015) downstream of the sfGFP coding sequence. Sequences for the strong (BBa_B0034), medium (BBa_B0032) and weak (BBa_B0033) RBSs were taken from the BioBrick part registry and the ‘dead’ RBS is the reverse compliment of BBa_B0034. Sequences for CrPV (AF218039), IAPV (NC_009025.1) and PSIV (AB006531) IGR IRESs with 18 nts of corresponding downstream coding sequences were obtained from GenBank (Summarized in Supplementary Table S1). We used the Salis Lab RBS calculator to ensure a strong RBS or an upstream start codon was not accidentally created during IRES mutagenesis and IRES scrambling (26).

### Cloning and site directed mutagenesis

Fluorescent reporter constructs were synthesized (Integrated DNA Technologies and Twist Biosciences) and subcloned into pSB3C5, a medium to low copy number plasmid. Pseudoknot (PK) mutations and deletions were introduced using the Quickchange™ method. All reactions were carried out using a T_Gradient_ (Biometra) thermocycler and resulting mutant plasmids transformed into electro competent BL21-Gold (DE3) cells (Agilent). The integrity of all constructs and PK mutations were confirmed by sequencing (Genewiz).

### Cell growth

50 ml of *E. coli* BL21-Gold (DE3) cells containing fluorescent constructs were grown in LB media to mid log phase (0.5 OD_600 nm_) at 37**°**C with shaking (200 rpm) in 125 ml Erlenmeyer flasks and expression induced with isopropylthio-β-galactoside (IPTG, 1 mM final concentration). Cells were then harvested at distinct time intervals (fluorescent time courses) or grown for three hours before being analyzed by flow cytometry.

### Flow cytometry

Cells were pelleted, washed twice with and subsequently resuspended in FACSFlow™ (BD Biosciences), and kept on ice until cytometric analysis. Flow cytometry was performed on a BD FACSAria Fusion cell sorter (488 nm excitation, observing sfGFP fluorescence in the FITC channel) and data analysis performed on Flowjo software (Flowjo, LLC). All flow cytometry was performed in biological triplicate, collecting 100 000 events per replicate.

### sfGFP immunoblotting

Whole cell lysate or 5 μl of PURExpress^®^ (New England BioLabs) reaction was loaded onto a nitrocellulose membrane (Pall Corporation) using a Biodot SF microfiltration apparatus (BioRad) and the presence of sfGFP was detected using an anti-GFP antibody (Abcam, ab6556) and a peroxidase conjugated secondary antibody (Sigma, A0545). Chemiluminescence from three biological replicates was quantified using an Amersham Imager 600 (GE healthcare).

As a secondary check regarding the effect the maturation time of the sfGFP has on signal generation, we used the GFP specific antibody to probe protein levels during the expression time course, which confirmed that our live cell fluorescence assay was accurately reporting protein levels and not variations in the sfGFP maturation times (Supplementary Figure S1).

### RT-qPCR

Total RNA from three biological replicates was extracted from *E. coli* using an EZ-10 total RNA purification kit (Bio Basic) and the integrity/purity confirmed using formaldehyde agarose gel electrophoresis and *A*_260_/*A*_280_ ratio (Biodrop). Using 100 ng of total RNA and the respective reverse primers (IDT) (sfGFP 5′-GATAACGAGCAAAGCACTGAAC-3′ and cysG 5′-ATGCGGTGAACTGTGGAATAAACG-3′) cDNA was generated using qScript cDNA Supermix (Quanta Biosciences) according to manufacturer's specifications. Quantitative PCR was performed according to manufacturer's specifications on a StepOnePlus Real-Time PCR System (Thermo Fisher) using PerfeCTa^®^ SYBR^®^ Green SuperMix (Quantabio) with the corresponding forward primers (IDT) (sfGFP 5′-GGTGACGCAACTAATGGTAAAC-3′ and cysG 5′-TTGTCGGCGGTGGTGATGTC-3′) and the above reverse primers. All sfGFP mRNA threshold values were scaled relative to the accompanying cysG reference mRNA threshold values to account for differences in cDNA input.

### sfGFP degradation assay

50 ml of *E. coli* BL21-Gold (DE3) cell containing fluorescent constructs were grown in LB media to mid log phase (0.6 OD_600 nm_) at 37**°**C with shaking (200 rpm) in 125 ml Erlenmeyer flasks in the presence of IPTG (1 mM final concentration). 50 ml of cells were pelleted and washed twice in AB minimal media before being resuspended in 50 ml of AB minimal media and incubated at 37**°**C with shaking (200 rpm) in 125 ml Erlenmeyer flasks (27). Cells were then harvested at specific time intervals and sfGFP measured by flow cytometry (as above). The OD_600 nm_ was constant at ∼0.6 over the course of the experiment ensuring cells were not actively dividing. No variations in sfGFP levels or degradation rates (Supplementary Figure S2) could be detected for the different constructs; in particular, the decay rate was so slow (sfGFP was stable over numerous days) that sfGFP degradation is negligible over the time of our experiments.

### RNA in vitro transcription, [^32^P] labelling and purification

DNA templates for *in vitro* transcription were generated by PCR using plasmids containing wild type IRESs, mutant IRESs, or control RNAs (Supplementary Tables S1 and S2). The obtained DNA was used in subsequent *in vitro* transcription reactions, and the resultant RNA purified by nucleic acid spin column (Bio Basic). The purity and homogeneity of the RNA was assessed by urea PAGE and *A*_260_/*A*_280_ ratio (BioDrop μlite, BioDrop).

Five hundred nanograms of IRES RNA in water was unfolded by heating to 95°C for 2 min before being snap cooled on ice. RNA was then dephosphorylated by incubating at 37°C with Shrimp Alkaline Phosphatase (0.001 U/μl final concentration, Fermentas) for 60 min. Two hundred and fifty nanograms of dephosphorylated RNA were incubated with T4 polynucleotide kinase (0.5 U/μl final concentration, Fermentas) and 1.5 μl of [^32^P]-γ-ATP (30 μl total reaction volume) for 60 min at 37°C. To quench the reaction 1.5 μl of 0.5 M EDTA pH 8.0 was added and the reaction subsequently heated to 75°C for 10 min before the RNA was purified via EZ-10 Spin Column RNA Cleanup and Concentration Kit (Bio Basic).

### Purification of prokaryotic and eukaryotic ribosomes

Prokaryotic 70S ribosomes and 30S ribosomal subunits were purified from *E. coli* MRE600 as per Becker *et al.* (28,29). Eukaryotic 40S ribosomal subunits were purified from HeLa cells (National Cell Culture Laboratory) as previously described (30).

### Removal of ribosomal protein S1 from 30S subunits (30S^–S1^ subunits)

30S ribosomal subunits were diluted tenfold in a high-salt dissociation buffer (20 mM Tris–HCl pH 7.5, 10 mM MgCl_2_, 60 mM KCl, 1 M NH_4_Cl and 1 mM DTT). The mixture was incubated at 37°C for 10 min before being added to poly(U) (Sigma Aldrich, P8563) and incubated at 4°C for 1 h with gentle inversion. The mixture was centrifuged at 500 × g for 5 min, and the supernatant collected. The 30S^–S1^ subunits were pelleted via ultracentrifugation with a Sorvall S55-S swinging-bucket rotor ultracentrifuge (Thermo Scientific) at 55 000 rpm, at 4°C for 24 h and resuspended in TAKM_5_ (50 mM Tris–HCl pH 7.6, 70 mM NH_4_Cl, 30 mM KCl, 5 mM MgCl_2_) to a concentration of ∼15 μM. S1 removal was confirmed via SDS-PAGE and mass spectrometry (U of L Mass Spectrometry Facility).

### Nitrocellulose filtration assays

Radio-labeled RNA (50 nM final concentration) in TAKM_5_ buffer was heated to 95°C for 10 min and slow cooled to room temperature. RNA was then incubated with increasing amounts of ribosomal subunits/ribosomes for 15 min at 37°C before being rapidly filtrated through a cellulose nitrate membrane filter (0.2μm, GE Healthcare). The cellulose nitrate membranes were washed with 1 ml of cold TAKM_5_ buffer and placed into 10 ml of EcoLite (+) scintillation cocktail (MP Bio), vortexed for 30 s, and subsequently incubated at room temperature for 30 min, followed by vigorous mixing for 30 s. The retained radioactivity was quantified by scintillation counting (Tri-carb 2810 TR LSA, Perkin Elmer). To ensure our system was able to replicate previously reported data we also performed binding assays with HeLa 40S subunits. Consistent with previous work the WT CrPV IRES bound the 40S with a *K*_D_ of ∼14 nM, while disruption of PK1 had no effect on 40S binding and disruption PK1 and PK3 in combination abolished 40S binding (Supplementary Table S3) (9,31).

### RNA fluorescence titrations

Purified WT CrPV IGR IRES RNA was labelled at the 3′ end with pyrene as per Keffer-Wilkes *et al.* (32). Labeled RNA (50 nM final) in TAKM_5_ was heated to 95°C for 10 min and slow cooled to room temperature. RNA was then incubated with increasing amounts of ribosomal subunits/ribosomal protein S1 before being excited at 341 nm. Ribosomal protein S1 was a generous gift from J.L.E. Heller and J.R.J. Vigar and was purified as previously described (33,34). The peak fluorescence at 391 nm was recorded and plotted as a function of increasing 30S or S1 concentration.

### Circular dichroism (CD) spectroscopy

Circular dichroism was performed on a Jasco J-815 CD Spectrometer using a 1s integration time over 200–320 nm (35). WT CrPV IRES RNA (∼150 nM) was folded as described previously (see nitrocellulose filtration assay) before being subjected to CD spectroscopy. Increasing amounts of ribosomal protein S1 was added, incubated at 37°C for 15 min before being scanned again. Ribosomal protein S1 alone was measured at each concentration and that data was subtracted from the RNA/protein mixture to ensure only RNA signal was being observed. All data were recorded 5 times, average traces are plotted.

### Statistical Information

For all statistical analyses *n* = 3 unless otherwise stated.

- A) *Flow cytometry*

Flow cytometry was performed as described in methods description. Mean fluorescence was calculated using FlowJo software (FlowJo, LLC). Standard deviation and relative significance (*t*-test, two tailed) of each data set was calculated using Microsoft Excel.

- B) *Immunoblot*

Immunoblot intensity was determined using the ImageJ gel analysis package (36). Mean intensity and standard deviation were calculated using Microsoft Excel.

- C) *RT-qPCR*

Threshold levels were generated by the StepOnePlus Real-Time PCR System and mRNA levels were calculated using the values generated from the StepOnePlus software (Applied Biosystems) and Microsoft Excel.

## RESULTS

**Figure 1.**
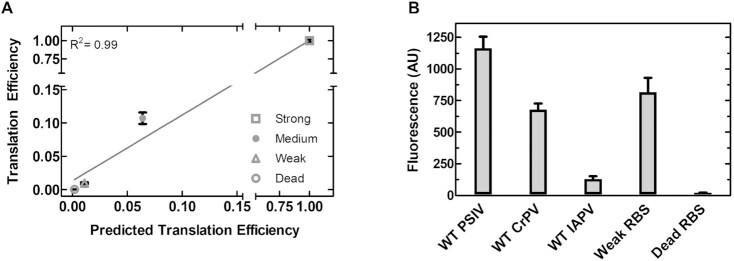
IGR IRES translation is weak compared to canonical ribosome binding sites. (**A**) Correlation of predicted and measured translation efficiency of standardized RBSs. Translation efficiency predicted using the Salis lab RBS calculator (26), translation efficiency measured by flow cytometry. Mean values of three biological replicates are plotted. (**B**) Translation efficiencies of the WT IGR IRESs compared to standardized RBSs. Translation efficiency measured by flow cytometry, mean values of three biological replicates are plotted and error bars indicate one standard deviation.

To quantify translation efficiency (TE) in live *E. coli* we utilized a fluorescence-based reporter (superfolder green fluorescent protein (sfGFP)) assay for monitoring live cell fluorescence via flow cytometry. We opted to measure individual live cell fluorescence by flow cytometry to avoid potential averaging of distinct *E. coli* populations. To benchmark our reporter system, we selected three ribosome binding sites (RBS) (Strong B0034, Medium B0032 and Weak B0033) from the registry of standard biological parts (http://parts.igem.org) (37), as well as a ‘dead’ RBS (the reverse complement of B0034), to drive the expression of sfGFP (Supplementary Figure S3). Flow cytometry measurements correlate nearly perfectly (*R*^2^ = 0.99) with the expression strength predicted using the ribosome binding site calculator, demonstrating the sensitivity and wide range over which our single-cell assay can accurately report translation efficiency *in vivo* without interference by endogenous mRNA expression (Figure 1A) (26,38). Using this assay and by benchmarking different IRESs against well-characterized RBSs we are able to accurately measure their translation efficiency, allowing for the first time a direct comparison of IRES translation to the canonical system and to assess their ability to compete with endogenous mRNAs *in vivo* (Figure 1B).

### The IGR IRESs have translational efficiencies comparable to a weak RBS *in vivo*

**Figure 2.**
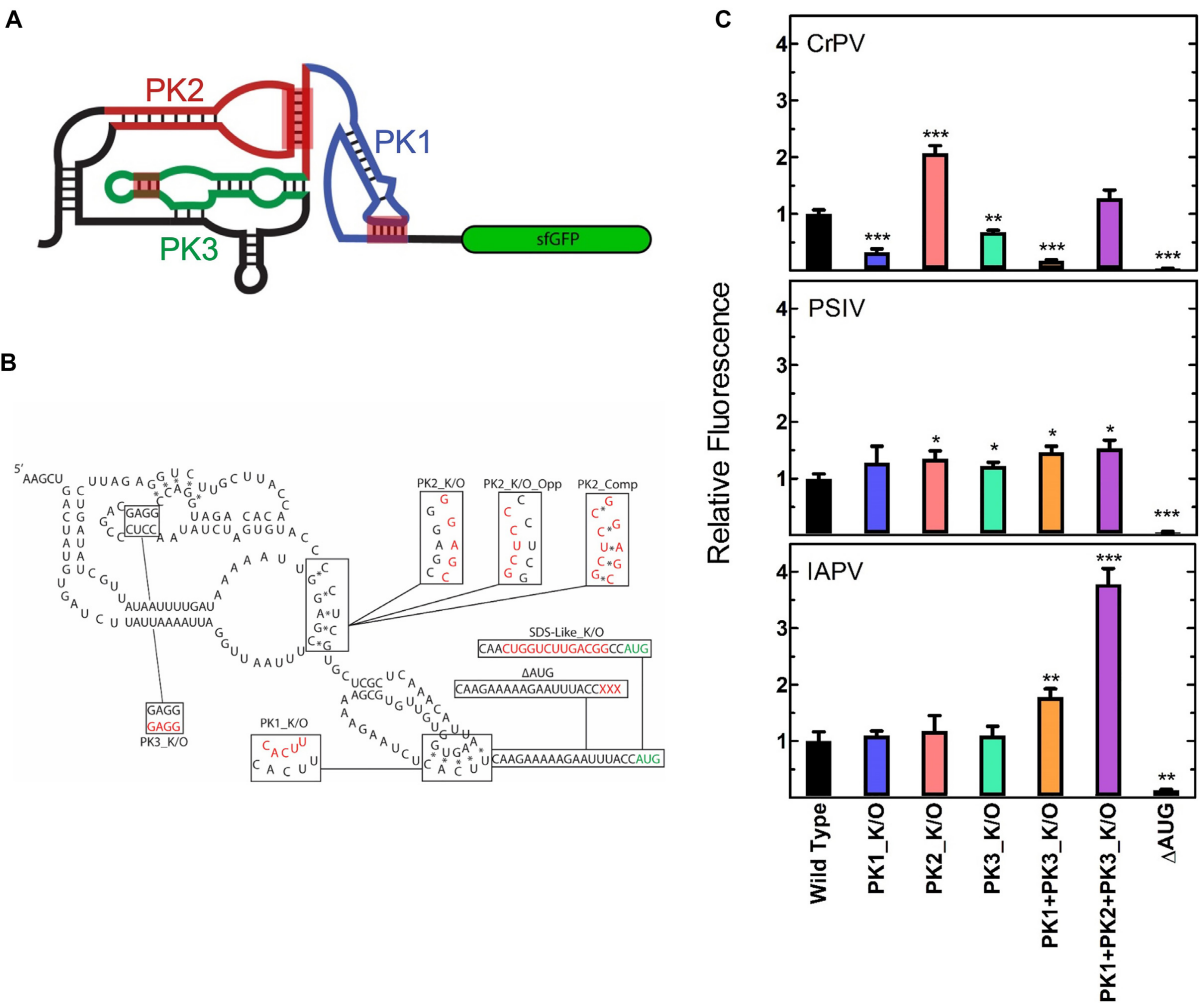
IGR IRES translation efficiency is independent of pseudoknot structure. (**A**) Cartoon representation of the monocistronic fluorescent reporter construct including the secondary structure of a generic IGR IRES, pseudoknots (PK) 1 (blue), 2 (red) and 3 (green) are indicated. (**B**) Secondary structure of the PSIV IRES with nucleotide substitutions for annotated mutations shown in red and the sfGFP start codon in green. (**C**) Translation efficiency of WT and IGR IRES variants measured by flow cytometry, mean values of three biological replicates are plotted relative to the respective WT IRES. For all panels error bars indicate one standard deviation. Constructs with statistically significant differences from WT are indicated (*P < 0.05, **P < 0.01, *** *P* < 0.001, **** *P* < 0.0001).

To ensure accurate representation of the IGR IRESs we selected two type I (*Plautia stali* intestine virus, PSIV and Cricket Paralysis Virus, CrPV) and one type II (Israeli Acute Paralysis Virus, IAPV) IGR IRESs. To accurately mimic the IRES expression we included 18 nucleotides of viral coding sequence downstream of the IRES to keep the initiation element in its native context and our reporter system (Figure 2A and B) consistent with previous studies (9,22). Finally, we opted for a monocistronic IRES construct to avoid potential translational coupling, as downstream translation and the intergenic RNA structure can be influenced by upstream translation (39,40). The obtained live-cell fluorescence data revealed that the translation efficiency of the type I IRES constructs (CrPV and PSIV) are comparable to the weak RBS while the type II (IAPV) is roughly an order of magnitude lower (Figure 1B). Interestingly, the same trend is observed for these IRESs in yeast and rabbit reticulocyte lysates, as the type I IRESs are more translationally efficient than type II IRESs (41,42). Deletion of the sfGFP start codon abolished translation in all IRES constructs, making them indistinguishable from cells with no fluorescent reporter (Figure 2C) and is consistent with previous work (22).

### Disruption of pseudoknots does not perturb type I or type II IGR IRESs activity *in vivo*

Although consistent with canonical bacterial translation, the observation that an AUG start codon is required for translation of the IRES-containing mRNAs raises the question regarding the role of the IRES structure in recruiting the ribosome and ultimately translation initiation. To investigate the detailed mechanistic role that the structured elements of the IRES play in its translational efficiency, we systematically disrupted conserved structural features (e.g. PKs, Figure 2B) present in the IGR IRESs (Summarized in Table 1) and measured the corresponding expression levels of sfGFP using flow cytometry. The PKs are critical for ribosome recruitment and translation initiation in eukaryotes. It has been previously demonstrated that altering the sequence of the IRES to disrupt the Watson–Crick–Franklin base pairs affects only the respective PK structure, leaving the rest of the IRES structures intact (9). Surprisingly, our measurements reveal (Figure 2C) that disruption of PK1, PK2, PK3 and combinations of these mutations have no negative effects on IAPV or PSIV IGR IRES mediated translation, in fact several of the constructs exhibit increased translation when compared to their wild type (WT) counterparts (Figure 2C). However, this is in contrast to previous work where disruption of PK2 reduced PSIV mediated translation ∼60% (22). In this context the CrPV IRES is unique as disrupting PK1 and PK3 decreases the translation efficiency by 70% and 30% respectively. When combined (PK1 + PK3_K/O) the translation efficiency decreases by 80% (Figure 2C). Unexpectedly, disruption of PK2 increased translation efficiency (∼100% relative to WT), and when combined with the other PK mutations (PK1 + PK2 + PK3_K/O), had a seemingly compensatory effect restoring activity to roughly WT level (Figure 2C).

**Table 1.** Relative *in vivo* translation efficiencies of IGR IRES constructs measured by flow cytometry. Mean values of three biological replicates are shown; error indicates one standard deviation

	PSIV	IAPV	CrPV
Wild Type	1.00 ± 0.08	1.00 ± 0.17	1.00 ± 0.07
PK1_K/O	1.28 ± 0.29	1.10 ± 0.08	0.32 ± 0.06
PK2_K/O	1.35 ± 0.13	1.18 ± 0.03	2.07 ± 0.13
PK3_K/O	1.22 ± 0.06	1.10 ± 0.16	0.67 ± 0.04
PK1+3_K/O	1.47 ± 0.11	1.78 ± 0.15	0.18 ± 0.01
PK1+2+3_K/O	1.54 ± 0.14	3.78 ± 0.28	1.29 ± 0.14
SD-like_K/O	0.04 ± 0.01	-	-
sfGFP ΔAUG	0.06 ± 0.01	0.14 ± 0.01	0.04 ± 0.01

The observation that disrupting pseudoknot structures often has no effect on IRES translation efficiency suggest that its tertiary structure is not required for the observed cross-kingdom expression activity of the IGR IRES. Complete deletion of PK elements from CrPV further supports this, as deletion of the highly structured PK2 and PK3 (ΔPK2/3) results in a 300% increase in translation efficiency while deletion of the relatively less structured PK1 (ΔPK1) abolishes translation (Supplementary Figure S4A). While these results do not align with a structure-based initiation mechanism, they can be interpreted through the lens of SD mediated translation of a highly structured 5′ untranslated region (UTR). In this context, removing a highly structured element (PK2/3) increases translation efficiency and placing this structured element near the start codon (by deleting PK1) hinders translation efficiency. To determine if the structure of the IGR IRESs offers any advantage over similar mRNAs with no such tertiary structure, we randomized the CrPV and IAPV sequences (while maintaining nucleotide composition) and assessed their translational efficiency using our *in vivo* fluorescence assay. Interestingly, the randomized CrPV and IAPV translation efficiencies are ∼200% and ∼600% greater than their WT counterparts (Supplementary Figure S4B and C), bringing CrPV translation efficiency to 200% of that of the weak RBS and elevates IAPV to the level of the weak RBS.

While the translational efficiencies of the IGR IRES PK variants do not agree with a structure-based interpretation, they correlate very well with the predicted translation efficiencies calculated using the Salis lab RBS calculator for bacterial mRNA expression strengths (PSIV *R*^2^ = 0.98, CrPV *R*^2^ = 0.76 and IAPV *R*^2^ = 0.89) (Supplementary Figure S5), suggesting canonical SD-based translation as the mechanism responsible for the observed expression of IRES containing mRNAs in bacteria. Together these results indicate that the tertiary structure of the IRES is not responsible for, but rather is inhibitory to, efficient translation. Therefore, we hypothesize that the IGR IRESs are being treated as large structured 5′ UTRs and translated via the canonical processes of the translation machinery that deal with the expression of structured mRNA rather than through molecular mimicry exploiting the conserved structural core of the ribosome (as is the case in eukaryotes).

### IRES translational efficiency is independent of bacterial growth phase

**Figure 3.**
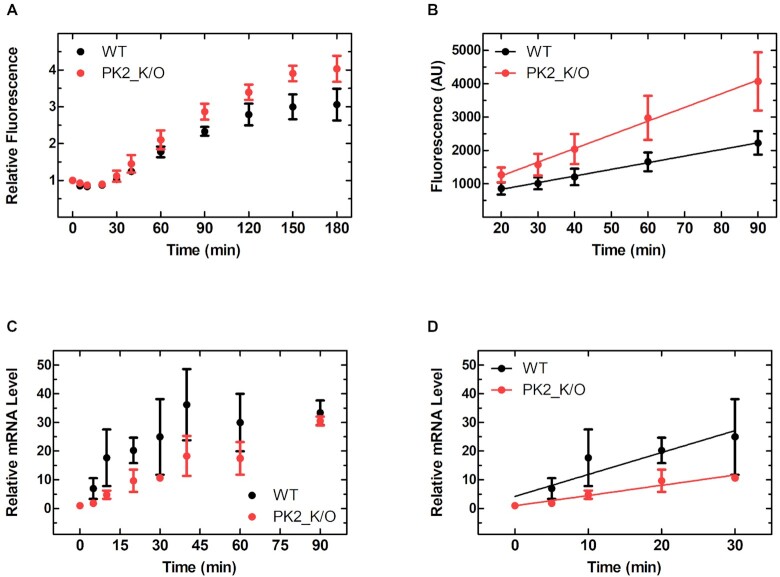
Differential PSIV IGR IRES translation efficiency is consistent over multiple growth phases and not an artifact of mRNA stability. (**A**) Relative fluorescence *in vivo* time course of *E. coli* containing PSIV IGR IRES constructs as measured by flow cytometry and (**B**) the linear portion of sfGFP expression in panel A. (**C**) Time course of relative mRNA level in live *E. coli* expression of PSIV IGR IRES constructs as measured by RT-qPCR. (**D**) Linear portion of mRNA expression from panel C. Mean values of three biological replicates are plotted; error bars indicate one standard deviation.

In our initial experiments we measured fluorescence at a single time point three hours post induction, shortly after entry into the stationary growth phase. However, it is possible that during times of increased competition for ribosomes (rapid growth) the structure of the IRES provides a kinetic advantage to the mRNA by transiently interacting with ribosomes in a structure dependent manner and increasing the local ribosome concentration. In order to determine if IRES translation activity is indeed due to canonical translation and if this is affected by the growth phase, we performed a detailed characterization of global IRES driven gene expression from mRNA transcription to protein degradation (Supplementary Figure S6). For this analysis, we selected the WT PSIV and PK2_K/O PSIV IRES constructs as they have different translation efficiencies in our initial experiments and in previous work (22). In agreement with our initial data, PK2_K/O reached a higher final fluorescence (Figure 3A) and the rate of sfGFP production during the exponential phase was 2-times greater for PK2_K/O (41.1 ± 1.4 min^–1^) than for WT (19.9 ± 0.6 min^–1^) (Figure 3B). This demonstrates that IRES translational efficiency is not affected by bacterial growth phase and again supports the notion that IRES tertiary structure is not responsible for translational activity.

### Disruption of IRES PK structure affects mRNA stability *in vivo*

While disruption of the PK structure has been shown previously to only affect local RNA structure (9), the PSIV PK2_KO mutation could be affecting the stability of the mRNA *in vivo*, lowering the abundance of the respective mRNA and as a consequence alter the measured translational efficiency. To assess mRNA accumulation and stability, we measured the total mRNA levels via qPCR on samples collected during the previous (*vide supra*) time course analysis. Interestingly, while mRNA levels were similar in the early stationary phase (Figure 3C) WT mRNA accumulated faster (∼2×) than PK2_K/O mRNA, suggesting that disruption of PK2 (PK2_K/O) indeed decreases mRNA stability (Figure 3D). This result also suggests that PK2_K/O has an even higher relative translational efficiency than reported by our fluorescence data when factoring in the reduced abundance of the respective mRNA.

### Location of the IRES element has no effect on translation initiation mechanism selection

**Figure 4.**
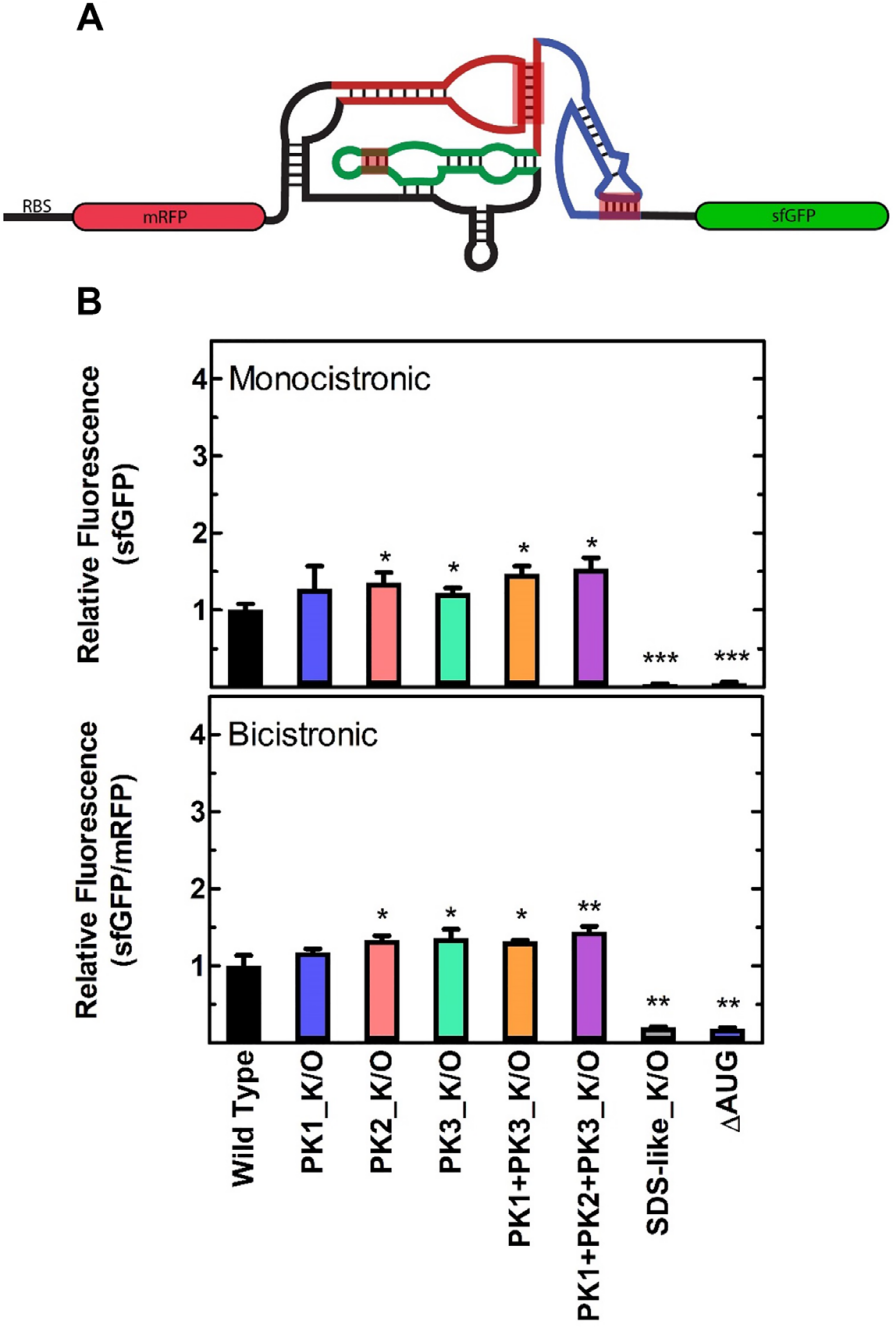
Relative PSIV IGR IRES translation efficiency is independent of its position in the mRNA. (**A**) Cartoon representation of the bicistronic fluorescent reporter construct including the secondary structure of a generic IGR IRES, pseudoknots (PK) 1 (blue), 2 (red) and 3 (green) are indicated. (**B**) Translation efficiency of the mono and bicistronic PSIV IGR IRES constructs measured by flow cytometry, mean values of three biological replicates are plotted relative to the respective WT IRES. Error bars indicate one standard deviation. Constructs with statistically significant differences from WT are indicated (*P < 0.05, **P < 0.01, ***P < 0.001, ****P < 0.0001).

In their natural context the IGR IRESs initiate translation in the intergenic region of a bicistronic viral genome (2,43). While canonical Shine-Dalgarno based translation, according to this definition, could be considered ‘IRES’ translation, it is possible that the structure of the IRES will be more efficient at promoting initiation if located between two genes as opposed to the monocistronic reporter we utilized (*vide supra*). To test this, we designed a bicistronic reporter construct whereby an upstream monomeric red fluorescent protein (mRFP) is translated via the weak RBS (B0033, the most similar to the WT PSIV IRES expression level) and the PSIV IRES drives the translation of sfGFP downstream (Figure 4A). The general design of this dual reporter construct is often used to validate IRES activity in eukaryotes and allows calculation of the mRFP/sfGFP ratio to control for intrinsic cellular noise. Subsequent analysis using flow cytometry revealed that the relative mRFP/sfGFP expression levels of the WT PSIV and PK variants are identical to the monocistronic expression levels (Figure 4B). These results demonstrate that the location of the IRES (5′ or internal) does not bias against a structure-based initiation mechanism and reaffirms our findings that the structure of the IRES is not essential for translation.

### IRES translation efficiency correlates with single stranded AG-rich sequences

We noticed a trend in our data that PK variants with increased translational efficiency almost always contained substitutions that introduced new single stranded (or liberated previously base-paired) AG-rich sequences upstream of the start codon, which have been shown to promote initiation (44). These single stranded sequences could be acting as ribosome standby sites, S1 binding sites, or simply facilitate the breathing of neighbouring RNA structures (45,46). As a simple check we introduced a compensatory mutation that re-establishes PK2 structure (PK2_Comp) in the PSIV and CrPV IRESs. This mutation returns PSIV translation efficiency to the wild type level and reduces the translation of the CrPV PK2_K/O IRES variant ∼70% (Figure 2B and S5A and B). However, the compensatory mutation alone (PK2_Opp), while disrupting PK2 structure, does not introduce a single stranded AG-rich sequence (Figure 2B) and interestingly has little to no effect on translation efficiency (Supplementary Figure S7A and B). These further support (at least for these particular variants) the notion that not the disruption of the PK structure, but rather the presence of a single stranded AG-rich sequence upstream of the start codon is important for its increased translation efficiency.

### Ribosomal protein S1 is required for efficient IRES translation

**Figure 5.**
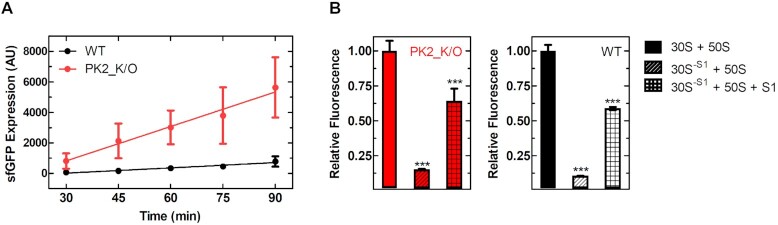
PSIV IGR IRES translation efficiency is strongly mediated by ribosomal protein S1. (**A**) Superfolder GFP production (*in vitro*) time course using the PSIV IGR IRES constructs and the PURExpress^®^ system. (**B**) Relative fluorescence of PSIV IGR IRES constructs using the PURExpress^®^ Δribosome system using complete ribosomes (30S + 50S), ribosomes lacking S1 (30S^–S1^ + 50S), and ribosomes lacking S1 supplemented with recombinant S1 (30S^–S1^ + 50S + S1). Mean values of three biological replicates are plotted, error bars indicate one standard deviation. Statistically significant differences between the WT and 30S^–S1^ samples and the 30S^–S1^ and 30S^–S1^+ S1 samples are indicated (***P < 0.001).

Our *in vivo* assays demonstrated that the structure of the IRES is non-essential and in fact even limits translational efficiency, suggesting that additional factors such as RNA helicases or chaperones (to resolve the structure of the IRES) might be required for their translation. To determine if cellular factors or the ribosome itself are responsible for this we measured the rate of IRES-mediated sfGFP production *in vitro* using the highly purified and reconstituted PURExpress^®^ system (47). The respective sfGFP synthesis time courses mirror our *in vivo* data, as the rate of sfGFP production is 6-times greater for PK2_K/O (75.3 ± 25.3 min^–1^) than for WT (11.5 ± 4.7 min^–1^) (Figure 5A). This supports the idea that the component responsible for the observed effect is present in the recombinant, purified, and reconstituted transcription and translation system, and also backs our earlier observation that the *in vivo* translation efficiency of the PSIV IRES PK2_K/O might be limited by decreased *in vivo* stability of the respective mRNA (Figure 3C and D) as the contributing nucleases are not present in the PURExpress^®^ system. Within the PURExpress^®^ system ribosomal protein S1 is the most likely candidate to resolve the IRES structure (48) as S1 binds to single stranded A-rich sequences and facilitates RNA unfolding essential for efficient canonical translation of mRNAs with structured 5′ UTRs in *E. coli* (46,48,49). If the IRESs are indeed being treated as large structured 5′ UTRs by the bacterial translation machinery, their translational efficiency will be reliant on S1. However, if specific interactions of the triple PK structure of the IRES with the ribosome are responsible for its translational efficiency, the absence of S1 should have little to no effect. To probe this hypothesis, we monitored IRES translation efficiency, using the PURExpress^®^ Δribosome kit supplemented with either complete ribosomes (30S + 50S) or ribosomes lacking S1 (30S^–S1^ + 50S). Both WT and PK2_K/O translation efficiencies were decreased ∼90% when S1 was not present (Figure 5B), likely due to the ribosome no longer being able to efficiently bind and unwind the highly structured RNA. To ensure this is a S1 specific effect and not due to the treatment of the ribosomes during S1 removal we supplemented stoichiometric amounts of recombinant S1 (30S^–S1^ + 50S + S1) resulting in a 60% recovery in activity for both constructs (Figure 5B). Additionally, recombinantly expressed ribosomal protein S1 binds the WT CrPV IGR IRES with an affinity roughly equivalent to the 30S ribosome (≤70 nM, Supplementary Figure S8), and is capable of unfolding the structure of the IRES in a concentration dependent manner (Supplementary Figure S9). Together this data demonstrates that S1 is indeed mediating the translation of the IGR IRESs by helping resolve their tertiary structure.

### IRES translation efficiency is dependent on a downstream SD-like sequence

If the IGR IRESs are being translated via the canonical translation initiation mechanism then the 30S ribosome will require a SD-like sequence adjacent to the sfGFP start codon for efficient translation (44). Interestingly, such a SD-like sequence is present in the viral coding region downstream of the PSIV IRES ((22) and Figure 2B). Altering this sequence to its reverse complement (SDS-like_K/O, Figure 2B) and leaving the IRES structure unchanged, abolishes translation in both the mono- and bicistronic reporters (Figure 4B) and further demonstrates that translation is proceeding via the canonical translation mechanism.

### Pseudoknot mutations do not perturb IGR IRES binding to the prokaryotic ribosome

While IRES PK structure is not responsible for the observed translation activity in prokaryotes, it is still possible that the IGR IRES is able to transiently interact with prokaryotic ribosomes in a structure specific manner that may be overshadowed by the canonical translation activity. In such a model disrupting the PKs structures will result in a reduced affinity of the IRES for the ribosome (as is the case for eukaryotic ribosome binding (9), Supplementary Table S3).

To investigate if the tertiary structure of the IGR IRES is responsible for prokaryotic ribosome binding, we determined the equilibrium binding constants (K_D_) for the CrPV and IAPV IRES-ribosome complexes using nitrocellulose filter binding. WT CrPV and IAPV IRESs RNA binds to the 30S subunit and the 70S ribosome with comparable affinities of ∼100 nM (Table 2). As expected, disrupting any of the PKs, individually or in combinations, has little to no effect on the affinity of either the CrPV or IAPV IGR IRESs for the 30S ribosomal subunit or the 70S ribosome (Table 2). This indicates that in contrast to the eukaryotic system (Supplementary Table S3) the IGR IRESs bind to the prokaryotic ribosome independently of their tertiary structure (9). This is consistent with the observation that prokaryotes do not have an equivalent to eS25, the eukaryotic ribosomal protein shown to be critical for ribosome binding and IRES activity (13,50,51). To ensure that the use of nitrocellulose filtration to determine the affinity for the respective mRNAs is not biasing against a transient population of structurally bound IRESs we also measured the affinity for the 30S ribosomal subunit using equilibrium fluorescence titrations with a pyrene labeled WT CrPV IRES, which resulted in a nearly identical affinity of ∼70nM (Supplementary Figure S10). Finally, for comparison we tested two native structured 5′ UTRs (*rpsO* and *sodB*) and found that their affinities to the 30S ribosomal subunit (*K*_D_ = 10 ± 5 nM and *K*_D_ = 13 ± 2 nM, respectively) and the 70S ribosome (*K*_D_ = 94 ± 14 nM and *K*_D_ = 47 ± 13 nM, respectively) are on the same order of magnitude as the IRESs (Supplementary Tables S4 and S5).

**Table 2.** Dissociation constants (*K*_D_) for CrPV and IAPV IGR IRES variants to 30S and 70S ribosomes as measured by nitrocellulose filter binding. Mean values of three biological replicates are shown; error indicates one standard deviation

**Construct**	**Ribosome**	**CrPV**	**IAPV**
		***K*_D_ (nM)**	***K*_D_ (nM)**
WT	70S	69 ± 7	50 ± 9
PK1_K/O	70S	92 ± 9	67 ± 3
PK2_K/O	70S	100 ± 13	59 ± 11
PK3_K/O	70S	90 ± 15	59 ± 4
PK1+3_K/O	70S	78 ± 10	65 ± 4
PK1+2+3_K/O	70S	79 ± 10	72 ± 14
WT	30S	110 ± 23	93 ± 7
PK1_K/O	30S	55 ± 11	87 ± 14
PK2_K/O	30S	74 ± 13	81 ± 25
PK3_K/O	30S	48 ± 5	103 ± 42
PK1+3_K/O	30S	52 ± 12	80 ± 28
PK1+2+3_K/O	30S	48 ± 7	73 ± 14

## DISCUSSION

The ability of an RNA molecule to ubiquitously initiate translation in a structure dependent manner is an exciting prospect for a number of applications including bioengineering and synthetic biology. While leaderless mRNAs have been described in all three domains of life (52), the PSIV IGR IRES would be the only RNA structure capable of initiating translation across domains and is currently the only example of structure based initiation described in prokaryotes (22). The proposed hybrid model of IRES mediated translation initiation in *E. coli* requires the IRES to first interact with the prokaryotic ribosome (in a structure-dependent manner) before it moves downstream to bind the RBS and initiate protein synthesis (22,23). This raises a number of interesting questions: do other examples of structure-based initiation exist in bacteria? How does the PSIV IGR IRES manipulate the bacterial ribosome? Is the ability to translate across domains of life an evolutionary conserved feature of all the IGR IRESs or is it specific to PSIV? In order to better understand this phenomenon, we utilized a dual pronged approach to benchmark IGR IRES translation efficiency (*in vivo* and *in vitro*) and ribosome binding (*in vitro*). We opted to measure several IGR IRESs as it has been shown previously that the IGR IRESs from different viruses have unique translational efficiencies (41,53). Interestingly our initial results demonstrated that disruption of the conserved PK elements does not consistently reduce IGR IRES translation efficiency and conversely often significantly increases translation efficiency. These results do therefore not support the previously proposed model of IGR IRES mediated translation in *E. coli* and is at odds with previously published data where disruption of PK2 (and variants with a PK2 disruption) lead to a decrease in PSIV IGR IRES mediated translation (22). We have ruled out the position of the IRES in the mRNA as the cause. However, it is possible that the reduced mRNA stability we observed for the PK2_K/O variant is more pronounced in a different mRNA context (sfGFP vs Luciferase coding sequences) which could easily explain the lower translation efficiency given our observation that mRNA stability is sensitive to the presence of single stranded regions. Unfortunately, no mRNA stability information is provided in the previous report (22).

**Figure 6.**
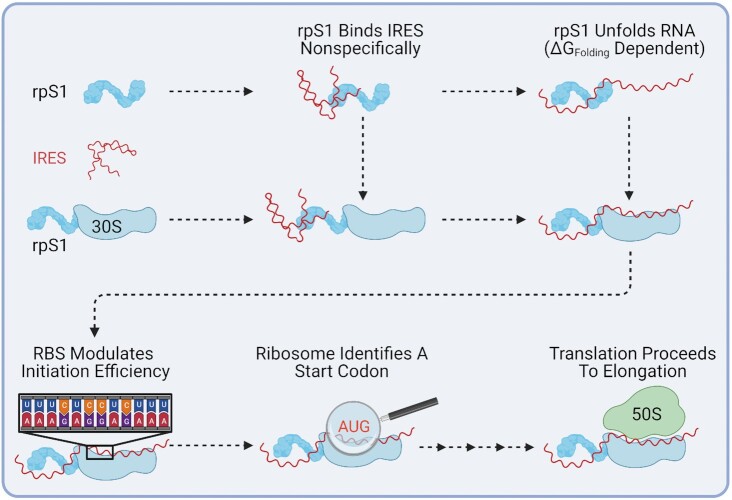
Ribosomal protein S1 mediates IGR IRES translation in *E. coli*. Ribosomal protein S1 non-specifically binds mRNA and assists in unfolding the mRNA structure. Next, the base pairing between the Shine-Dalgarno and anti-Shine-Dalgarno occurs and the start codon is located. Finally, upon 50S subunit recruitment the ribosome proceeds to translation elongation. For ease of view initiation factors and initiator tRNA have been omitted.

IRES variant translation efficiency data do correlate well with the mRNAs predicted free energy of folding (*R*^2^ = 0.61, Supplementary Figure S11) using mFOLD (54) and the predicted translation efficiency using the Salis lab RBS calculator (Supplementary Figure S5), respectively (26). This suggests that the IGR IRESs are being translated as structured mRNA through the canonical system rather than through a hybrid structure-based mechanism (Figure 6), highlighting the robustness of *E. coli*’s translation initiation machinery. A mechanism supported by recent findings that ribosomes with altered anti-SD sequences (incapable of base-pairing to canonical SDs) are able to initiate at the correct codons, suggesting translation start sites are determined by inherent mRNA features such as upstream A-rich sequences and lower levels of surrounding mRNA structure (44,55).

While taken together, our data indicates that IGR IRES structure is not required and in fact even inhibitory to translation in *E. coli*, it is exceedingly difficult to prove that the IGR IRESs are not transiently interacting with prokaryotic ribosomes. In eukaryotes the IGR IRES uses its triple pseudoknot structure to bind the conserved core of the ribosome and manipulate it into initiating translation in the P site without an initiator tRNA. While the same extent of manipulation does not occur in prokaryotes (an initiator tRNA and start codon are required) the IRES could be binding to the ribosome in a structure dependent manner. This claim is supported by the crystal structure of the IGR IRES from the PSIV bound to the *Thermus thermophilus* 70S ribosome (22) in which the IRES occupies the universally conserved tRNA binding sites similar to what is observed for related IRESs bound to the eukaryotic ribosome (14,56). However, there are marked differences which are discussed in the original report (22), the most significant is that only PK1 (tRNA–mRNA mimic) could be resolved using the density and the remaining PKs needed to be modeled, additionally PK1 was found bound to the P site, rather than the A site (22). Given that deacylated tRNAs are known to be stably bound and copurify with ribosomes in crystallography experiments (57) it's not unreasonable to assume that a tRNA–mRNA mimic could bind to the conserved core of the ribosome in a highly purified and reconstituted system without being physiologically relevant (24). Interestingly IRES–ribosome complexes were only detectable in *E. coli* lysate when the PSIV IRES had an 88nt tail and the elongation inhibitor Hygromycin B was present (22). It is impossible to differentiate using this assay if the ribosome is bound to the PSIV IRES and not the SDS-like sequence present in the tail, which is why in our binding experiments (Tables 2 and Supplementary Table S3, Supplementary Figures S8 and S10) we only included a small tail (8nts) after the IRES. Additionally, Hygromycin B is a ubiquitous inhibitor of elongation and acts through stabilizing tRNA binding to the ribosome (58). Considering the structural similarity of the IGR IRESs to a tRNA–mRNA duplex it is possible that Hygromycin B could artificially increase the affinity of the IRES for the ribosome. This is supported by the fact that Hygromycin B does not interfere with the ability of the PSIV IGR IRES to bind ribosomes but does suppress IGR IRES stimulated eEF2 GTPase activity and initial aa-tRNA binding to PSIV IGR IRES-ribosome complexes (59).

In agreement with previously published data (9,60) disruption of PK structure significantly reduced the affinity of the IGR IRESs for eukaryotic ribosomes (Supplementary Table S3). However, disruption of PK structure had either no effect or a significantly milder effect on prokaryotic ribosome binding for both CrPV or IAPV IGR IRESs (Table 2). Disruption of PK1 or PK2 for CrPV and PK1 or PK1 + PK3 for IAPV slightly reduced (10–15% and 4–8%, respectively) the affinity of the IRESs for the 70S ribosome. Most disruptions had no effect on 70S binding and which is a rather unlikely pathway for translation initiation *in vivo*, more physiologically relevant are the effects of structure disruption on the interaction of the IRES with the 30S subunit. Interestingly, all PK disruptions (with the exception of PK2) for CrPV and disruption of PK1 + PK2 + PK3 for IAPV increased the affinity (25–40% and 10%, respectively) for the 30S ribosomal subunit. This is in line with our *in vivo* data indicating that structure has an inhibitory effect on translational efficiency by reducing recruitment of the 30S. Together this suggests that the IGR IRES are being bound non-specifically by the prokaryotic ribosome (Figure 6) rather than through specific structure-based contacts.

Finally, while attempting to determine factors responsible for the translation efficiency of the IGR IRESs, we identified that IGR IRES translation is strongly dependent on ribosomal protein S1 (Figure 5B) and the presence of the SD-like sequence upstream of the start codon (Figure 4B). This is consistent with S1′s canonical role in host translation as it has already been shown to be essential for the translation of most endogenous *E. coli* mRNAs (61). During translation initiation S1 binds single stranded RNA in a sequence independent fashion (although preference for AU-rich regions has been reported (62,63)), a critical role as many bacterial genes do not contain Shine-Dalgarno sequences (48,61,63–65). Additionally, S1 binds RNA containing pseudoknots and has been shown to be essential for docking and unfolding of structured mRNAs on the ribosome (48,66). Previous studies have also reported that S1 can allow foreign mRNAs devoid of guanines (and therefore no SD sequence) from a plant infecting virus to form initiation complexes *in vitro* (64). It is tempting to imagine ribosomal protein S1 extending into solution and non-specifically binding mRNAs and ‘handing them over’ to the ribosome, increasing the local concentration in much the same manner as the ribosomal L7/L12 stalk for Elongation Factors Tu and G (67,68). Interestingly, in systems where mRNA structure can greatly influence translation (e.g. the PURExpress^®^ system) excess S1 (relative to ribosome concentration) has been shown to improve yield from structured mRNAs including the PSIV IGR IRES (69). This suggests that the critical activity of ribosomal protein S1 (i.e. RNA unfolding or potentially stabilizing unstructured mRNAs slowing degradation *in vivo*) may extend beyond ribosomal bound S1 to free cytosolic S1. Collectively, our data demonstrate that the translational activity of the IGR IRESs is not due to their three-dimensional structure and is primarily the result of the activity of ribosomal protein S1 and the overall robustness of canonical SD-dependent translation (Figure 6), closing the door on exploiting these IRES elements for the design of cross-kingdom mRNA-based bioengineering and synthetic biology.

## DATA AVAILABILITY

Data are available upon request.

## Supplementary Material

gkac697_Supplemental_File
