# Supplementary material for: The prokaryotic activity of the IGR IRESs is mediated by ribosomal protein S1
Source: Nucleic Acids Res. 2022 Aug 30;50(16):9355–67. doi: 10.1093/nar/gkac697 (PMC9458429; doi:10.1093/nar/gkac697)
Supplement: gkac697_Supplemental_File [file gkac697_supplemental_file.docx]

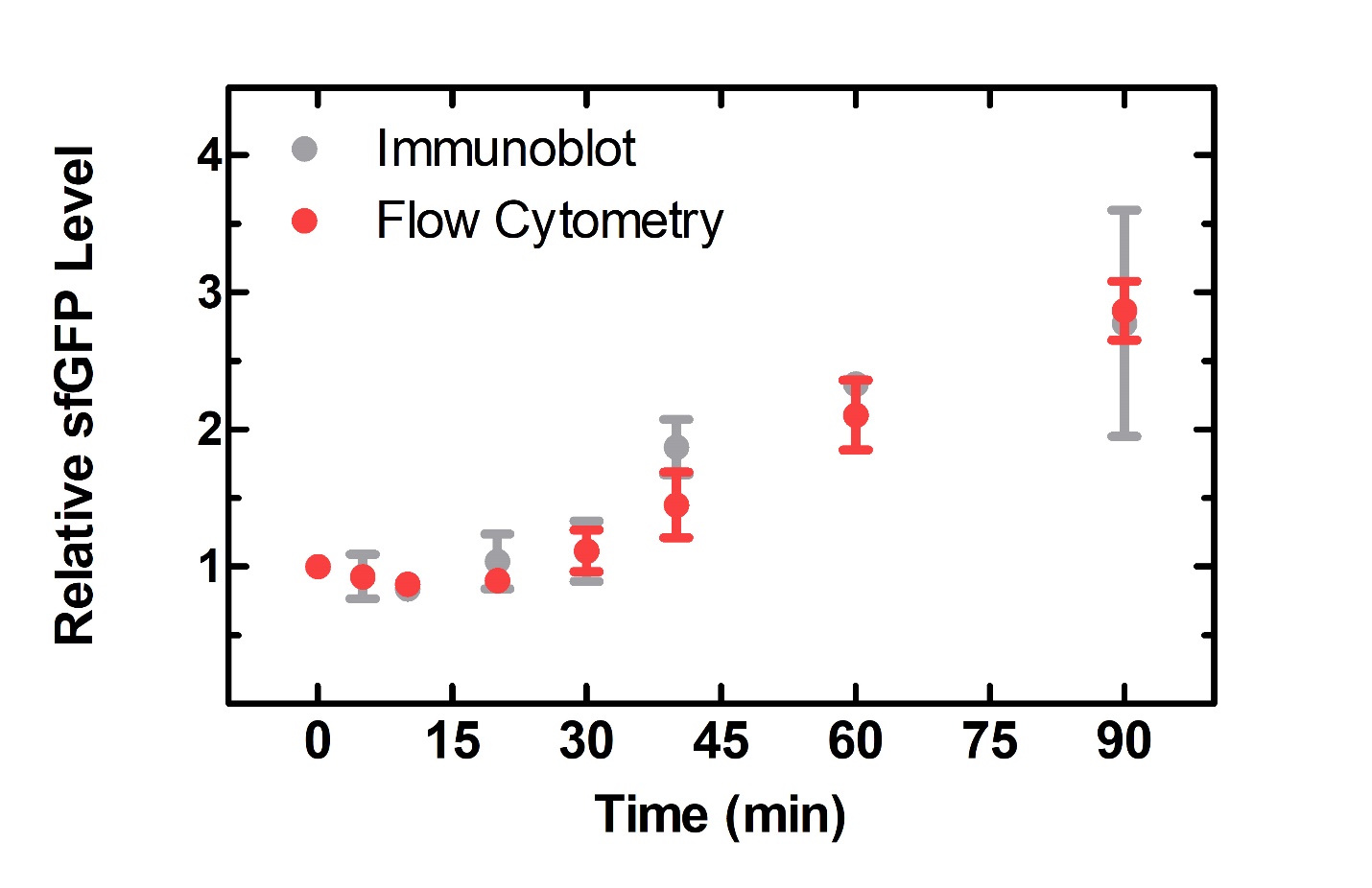


**Figure S1. Flow cytometry accurately reports the amount of sfGFP protein *in vivo*.** Relative fluorescence time course of *E. coli* containing PSIV IGR IRES constructs. Comparison between PK2_K/O flow cytometry data (fluorescence, red) and PK2_K/O immunoblot data (protein level, grey). Mean values of three biological replicates are plotted; error bars indicate one standard deviation.


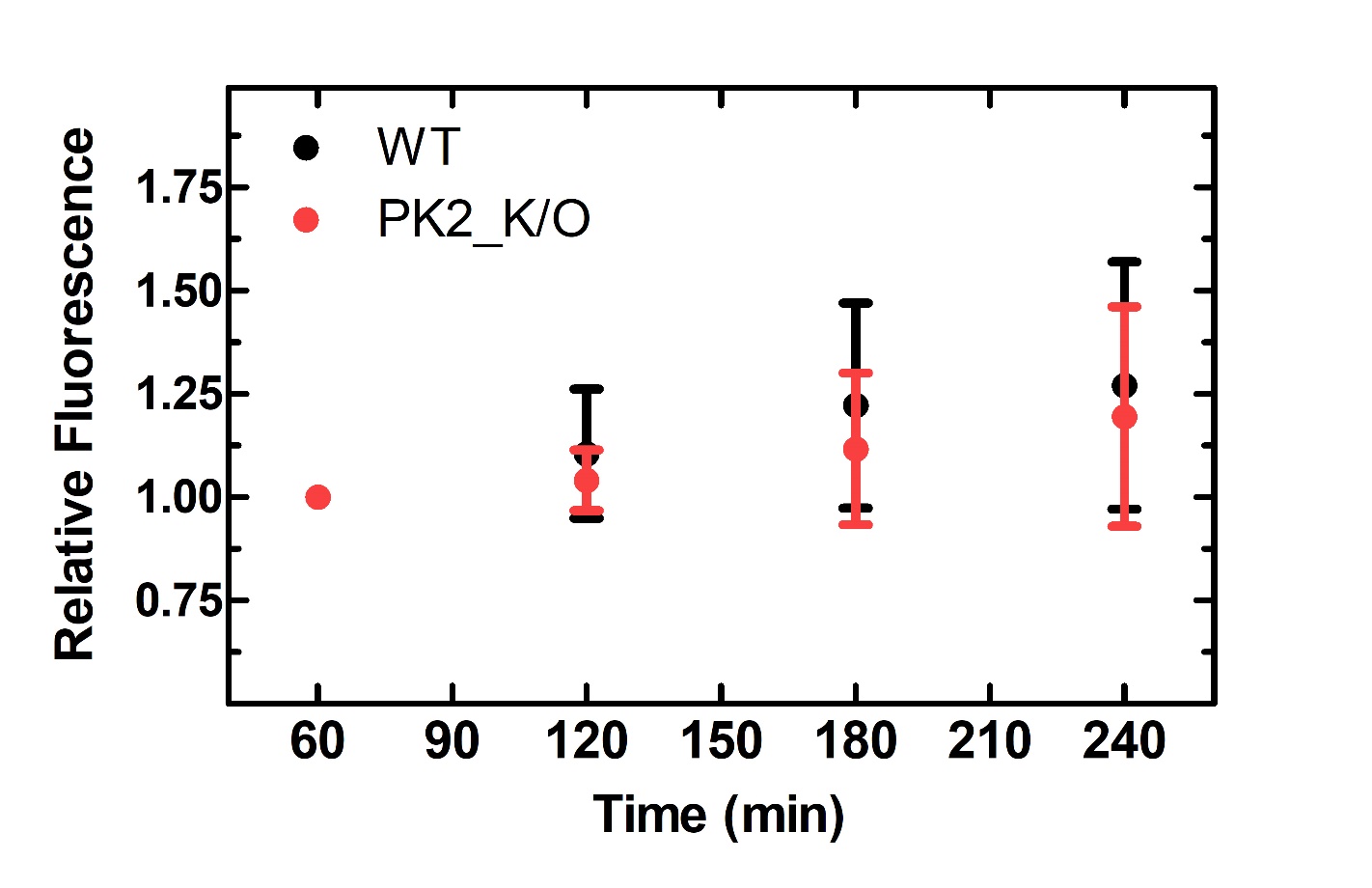


**Figure S2**. **Superfolder green fluorescent protein (sfGFP) is stable over multiple hours *in vivo***. Relative fluorescence time course of *E. coli* containing PSIV IGR IRES constructs post shift to minimal media measured by flow cytometry. Mean values of three biological replicates are plotted; error bars indicate one standard deviation.


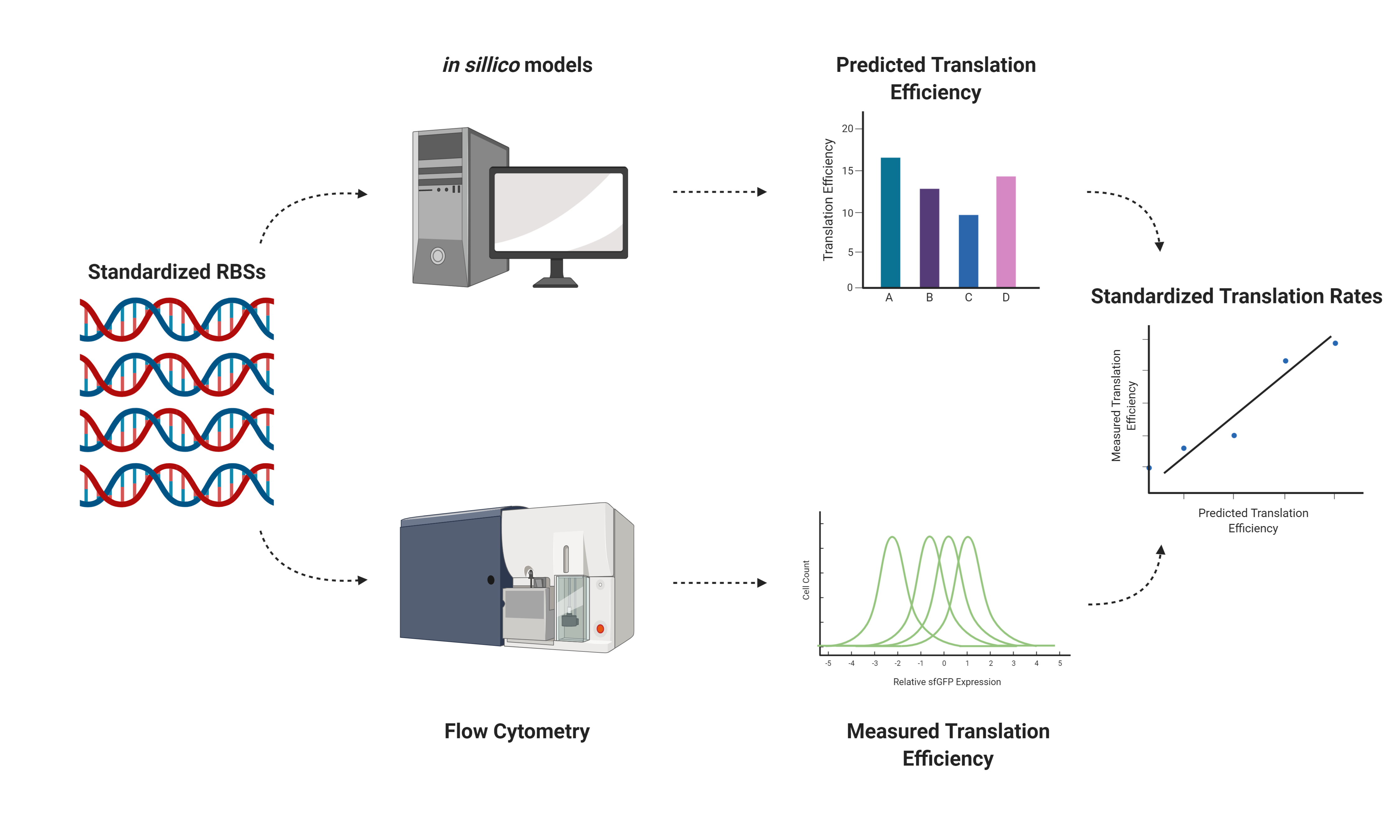


**Figure S3.  Novel real-time fluorescence-based single-cell translation assay for benchmarking IRES translational efficiency *in vivo*.** Using standardized ribosome binding sites, the translation efficiency of foreign translation elements can be benchmarked against canonical bacterial translation.


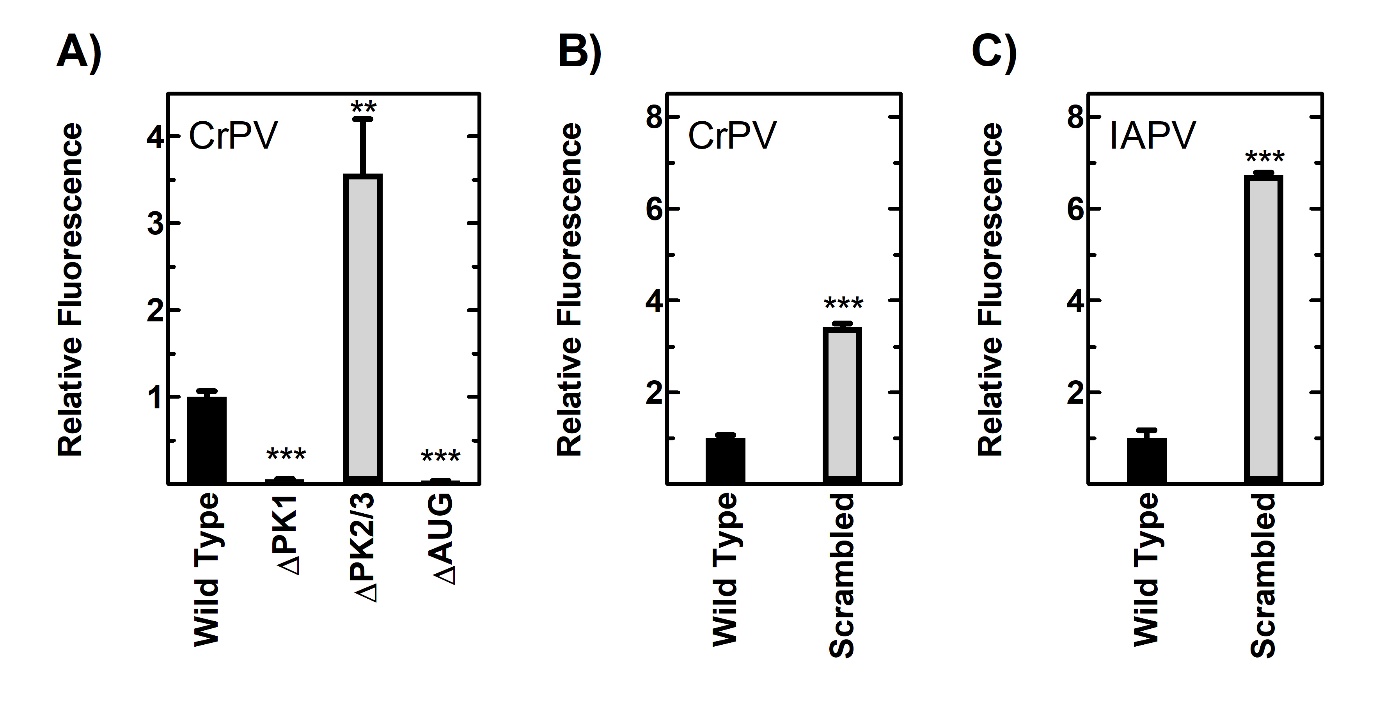


**Figure S4. Pseudoknot deletion and Scrambled IGR IRES variants have translation efficiency inconsistent with a structure-based mechanism.** Relative fluorescence of *E. coli* containing IGR IRES constructs. Translation efficiency measured by flow cytometry, mean values of three biological replicates are plotted relative to the respective WT IRES; error bars indicate one standard deviation. Constructs with statistically significant differences from WT are indicated (* = P < 0.05, ** = P < 0.01, *** = P < 0.001, **** = P < 0.0001).


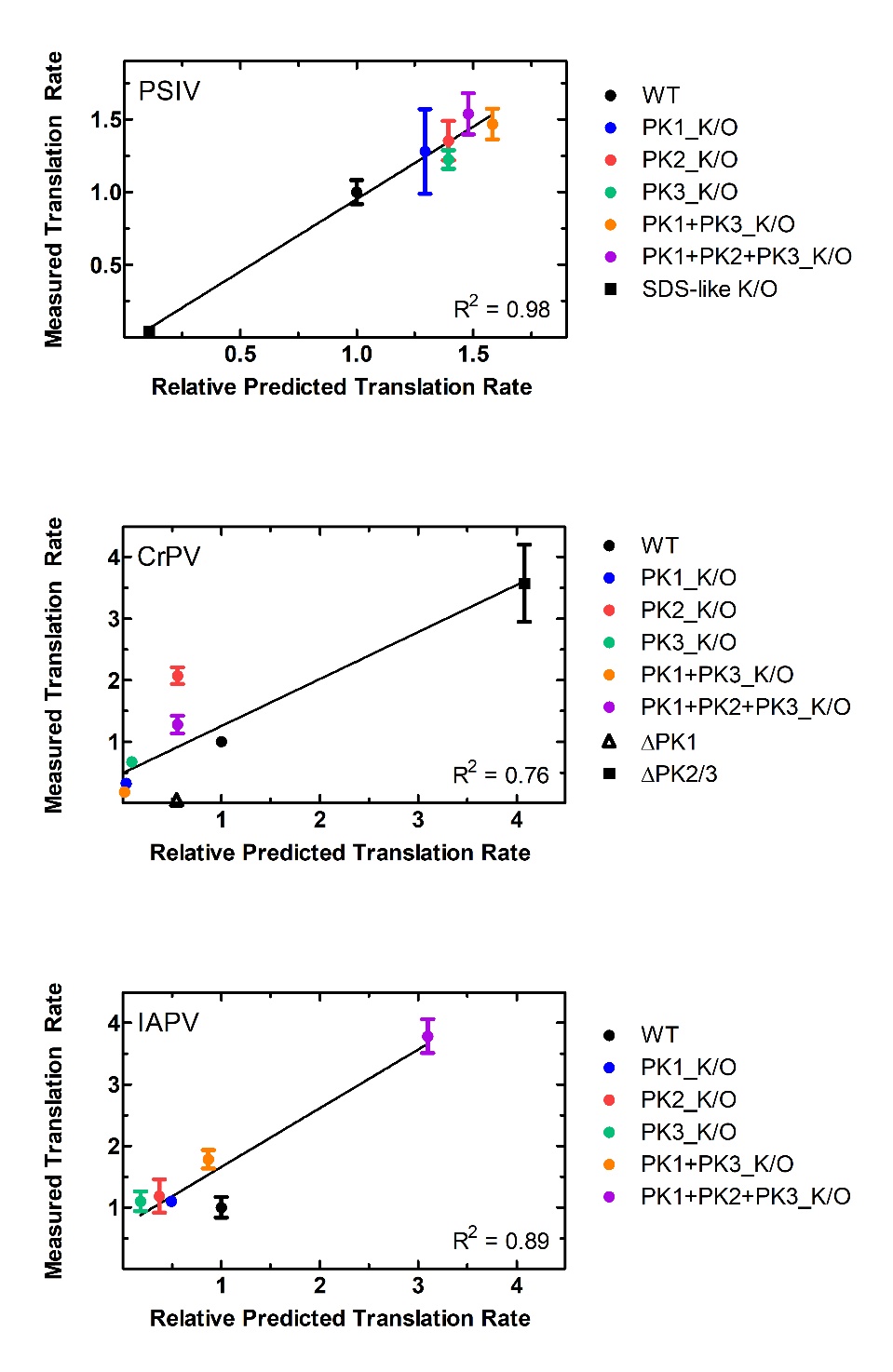


**Figure S5.** **Correlation of predicted and measured translation efficiency of IGR IRES constructs.** Translation efficiency predicted using the Salis lab RBS calculator, translation efficiency measured by flow cytometry. Mean values of three biological replicates are plotted; error bars indicate one standard deviation.


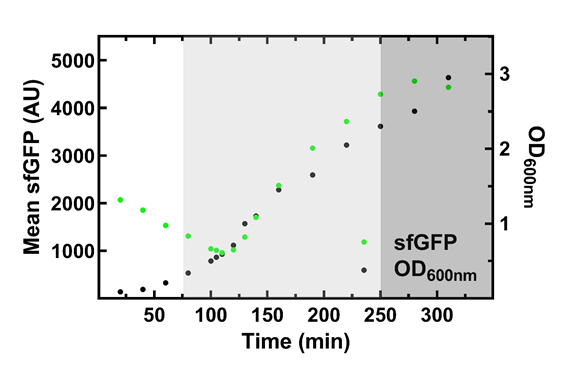


**Figure S6.** **Time course of sfGFP expression over several phases of cell growth.** Relative fluorescence *in vivo* time course (green) of *E. coli* containing the WT PSIV IGR IRES construct as measured by flow cytometry compared to the optical density at 600nm (black). Growth phases are indicated by the white (lag), light grey (exponential), and dark grey (early stationary) shading. Fluorescence decreased over the first 120 min as cells divide and dilute the sfGFP before expression is induced at 120min (0.6 OD 600nm) with IPTG.


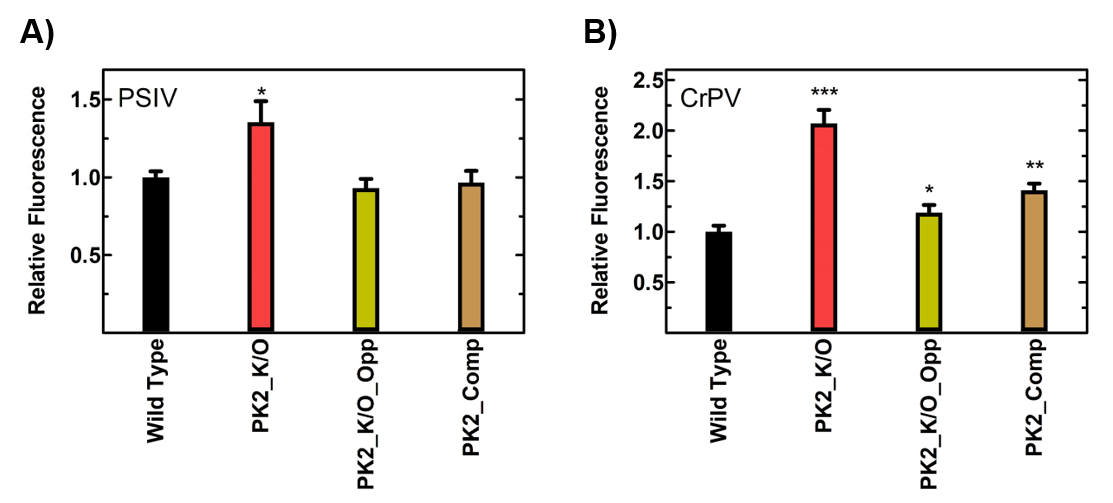


**Figure S7.  Compensatory mutation reduces PK2_K/O IRES translation efficiency**. Mean fluorescent values of three biological replicates obtained by flow cytometry are plotted relative to the respective WT IRES; error bars indicate one standard deviation. Constructs with statistically significant differences from WT are indicated (* = P < 0.05)


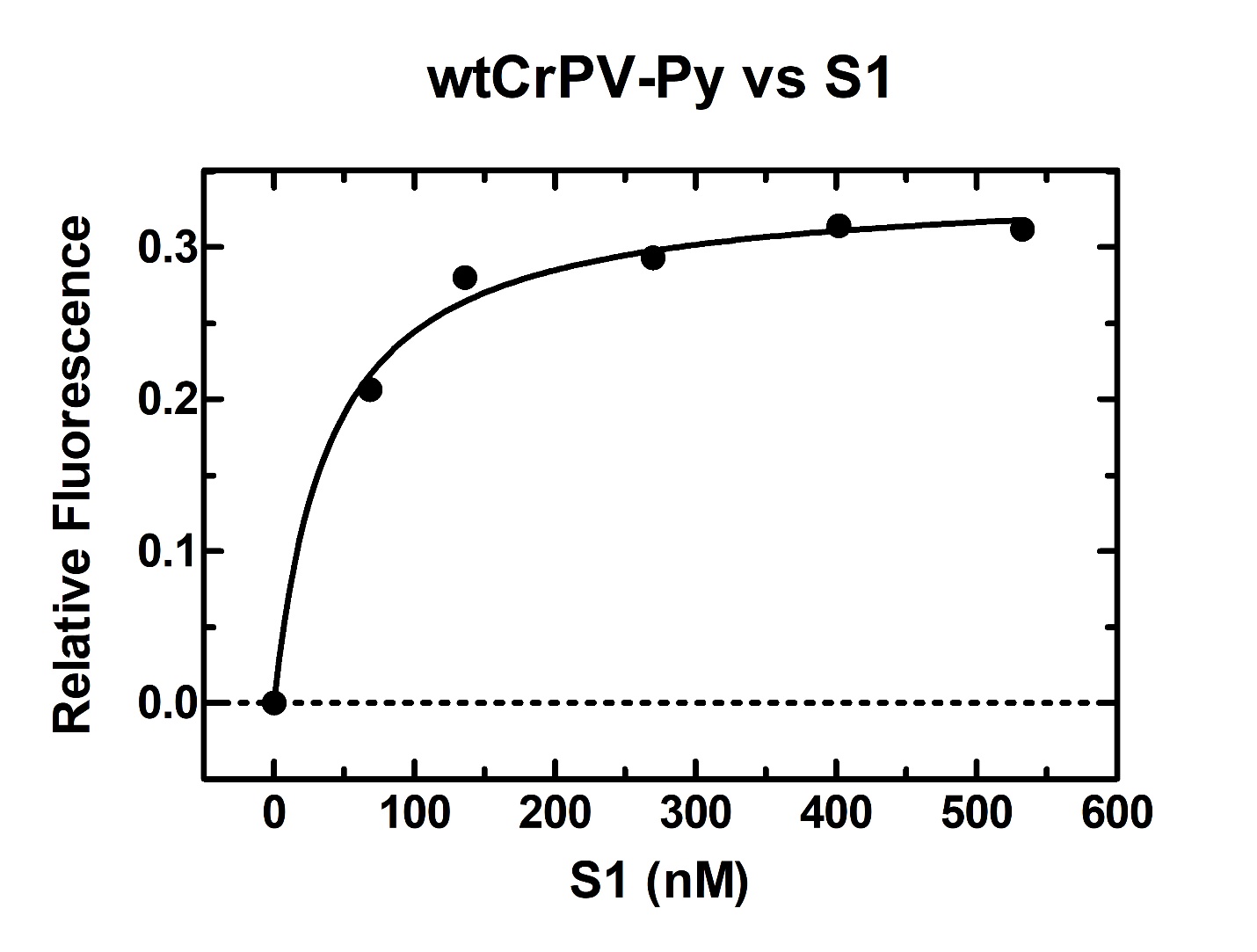


**Figure S8**.**The CrPV IGR IRES binds the ribosomal protein S1 with nanomolar affinity.** Titration of fluorescently labelled (pyrene) WT CrPV IGR IRES with ribosomal protein S1. Fluorescently labeled WT CrPV IGR IRES RNA is incubated with increasing amounts of ribosomal protein S1. Relative fluorescence emission at 391 nm is shown (λ_ex_ = 341 nm).


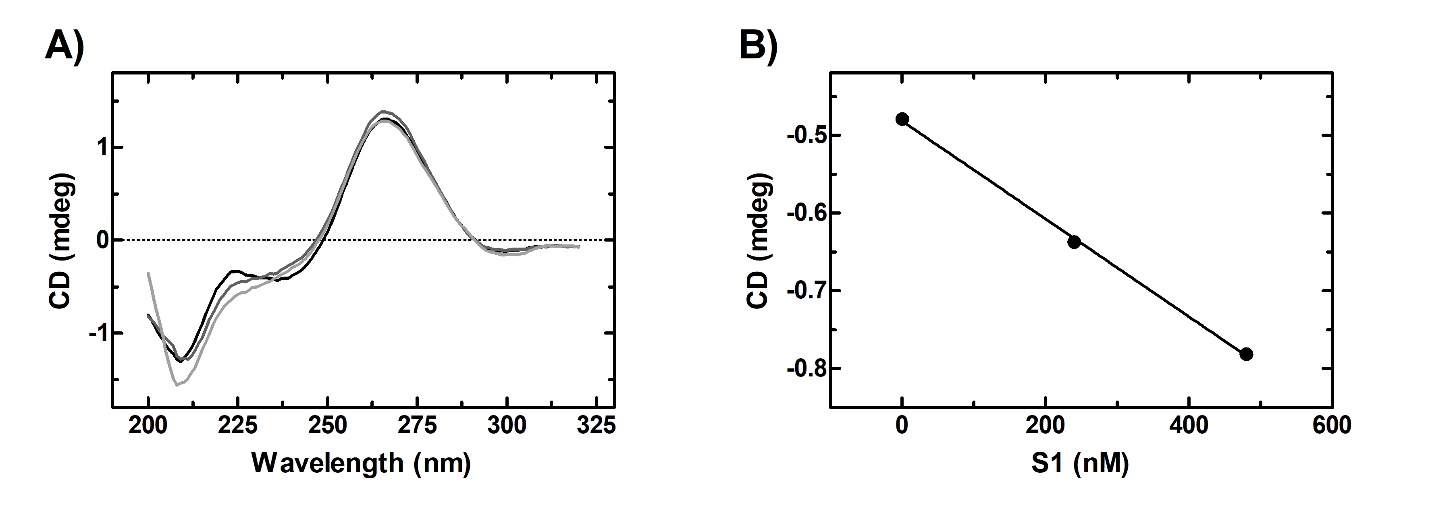


**Figure S9**.**Ribosomal protein S1 alters IRES RNA structure.** (A) Titration of WT CrPV IGR IRES with ribosomal protein S1 while measuring RNA structure via circular dichroism spectroscopy. Wild type CrPV IGR IRES RNA is incubated with increasing amounts of ribosomal protein S1, black trace (0 nM S1), dark grey trace (240 nM S1), light grey trace (480 nM S1). (B) Change in helical RNA structure at 220nm is plotted against S1 concentration.


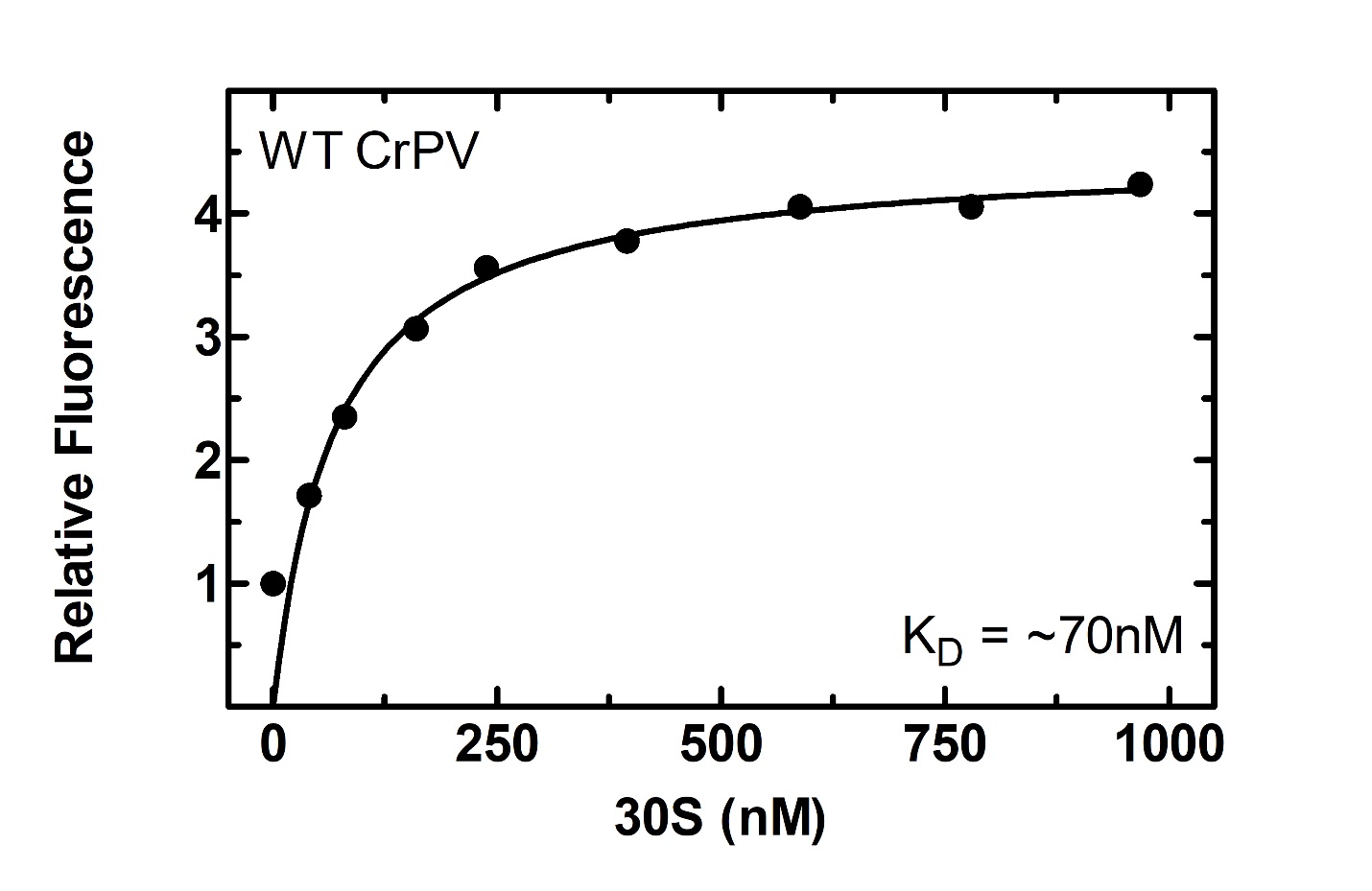


**Figure S10**.**The CrPV IGR IRES binds the 30S subunit with a nanomolar affinity.** Titration of fluorescently labelled (pyrene) WT CrPV IGR IRES with the 30S ribosomal subunit. Fluorescently labeled WT CrPV IGR IRES RNA is incubated with increasing amounts of 30S subunits. Relative fluorescence emission at 391 nm is shown (λ_ex_ = 341 nm).


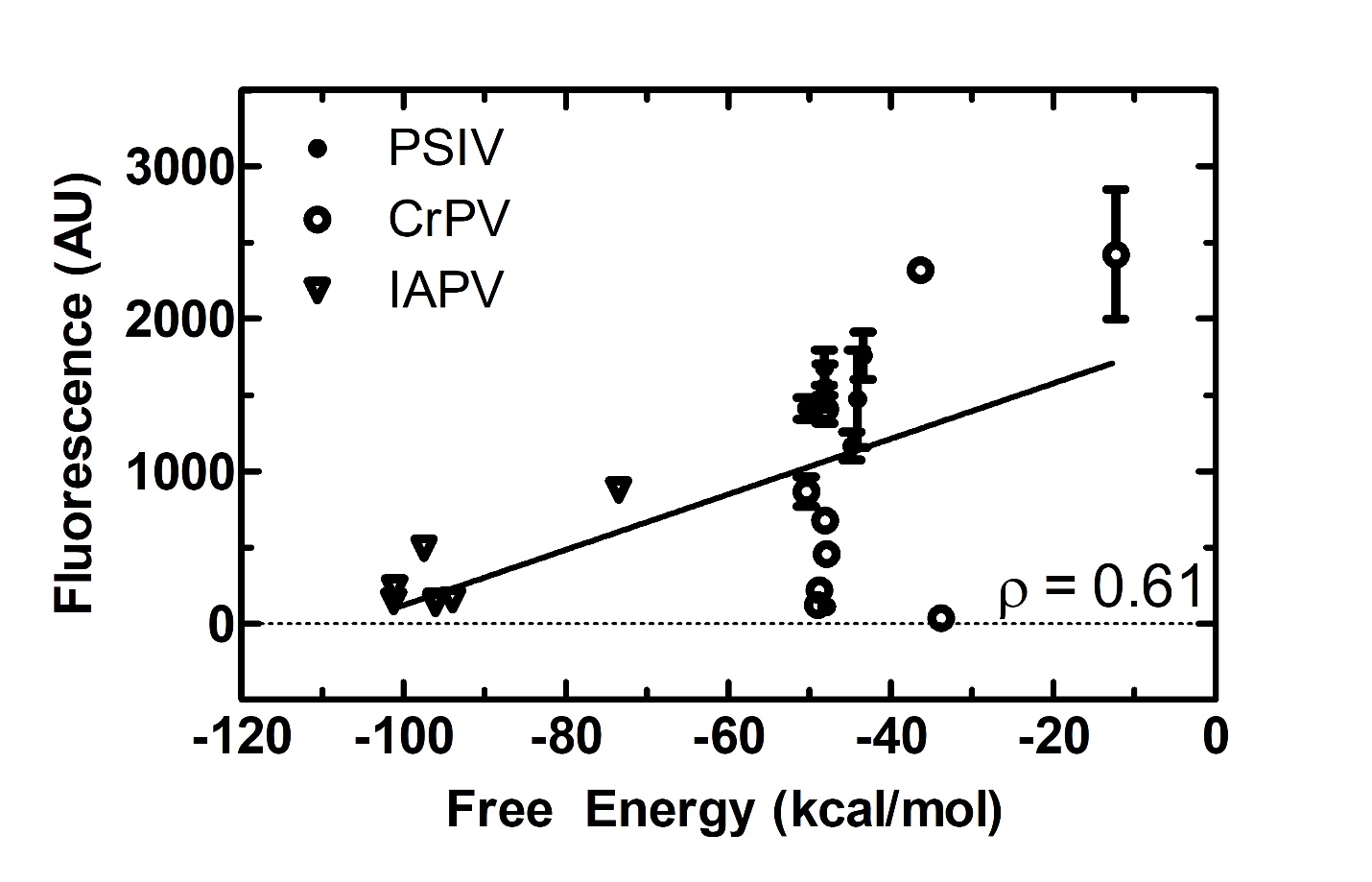


**Figure S11.** **Predicted free energy and translation efficiency of IGR IRES constructs are correlated.** Free energy predicted using mFOLD, translation efficiency measured by flow cytometry. Mean values of three biological replicates are plotted.

**Table S1.** DNA sequences of monocistronic and bicistronic* constructs.

| CrPV | Sequence Upstream from sfGFP |
| --- | --- |
| WT | 5'-AAAGCAAAAATGTGATCTTGCTTGTAAATACAATTTTGAGAGGTTAATAAATTACAAGTAGTGCTATTTTTGTATTTAGGTTAGCTATTTAGCTTTACGTTCCAGGATGCCTAGTGGCAGCCCCACAATATCCAGGAAGCCCTCTCTGCGGTTTTTCAGATTAGGTAGTCGAAAAACCTAAGAAATTTACCTGCTACATTTCAAGATAAA-3' |
| PK1_K/O | 5'-AAAGCAAAAATGTGATCTTGCTTGTAAATACAATTTTGAGAGGTTAATAAATTACAAGTAGTGCTATTTTTGTATTTAGGTTAGCTATTTAGCTTTACGTTCCAGGATGCCTAGTGGCAGCCCCACAATATCCAGGAAGCCCTCTCTGCGGTTTTTCAGATTAGGTAGTCGAAAAACCTAAGAAATTTAGGTGCTACATTTCAAGATAAA-3' |
| PK2_K/O | 5'-AAAGCAAAAATGTGATCTTGCTTGTAAATACAATTTTGAGAGGTTAATAAATTACAAGTAGTGCTATTTTTGTATTTAGGTTAGCTATTTAGCTTTACGTTCCAGGATGCCTAGTGGCAGCCCCACAATATCCAGGAAGCGGAGAGTGCGGTTTTTCAGATTAGGTAGTCGAAAAACCTAAGAAATTTACCTGCTACATTTCAAGATAAA-3' |
| PK3_K/O | 5'-AAAGCAAAAATGTGATCTTGCTTGTAAATACAATTTTGAGAGGTTAATAAATTACAAGTAGTGCTATTTTTGTATTTAGGTTAGCTATTTAGCTTTACGTTCCAGGATGCCTAGTGGCAGCCCGTGAATATCCAGGAAGCCCTCTCTGCGGTTTTTCAGATTAGGTAGTCGAAAAACCTAAGAAATTTACCTGCTACATTTCAAGATAAA-3' |
| PK1+PK3_K/O | 5'-AAAGCAAAAATGTGATCTTGCTTGTAAATACAATTTTGAGAGGTTAATAAATTACAAGTAGTGCTATTTTTGTATTTAGGTTAGCTATTTAGCTTTACGTTCCAGGATGCCTAGTGGCAGCCGGTGAATATCCAGGAAGCCCTCTCTGCGGTTTTTCAGATTAGGTAGTCGAAAAACCTAAGAAATTTAGGTGCTACATTTCAAGATAAA |
| PK1+PK2+PK3_K/O | 5'-AAAGCAAAAATGTGATCTTGCTTGTAAATACAATTTTGAGAGGTTAATAAATTACAAGTAGTGCTATTTTTGTATTTAGGTTAGCTATTTAGCTTTACGTTCCAGGATGCCTAGTGGCAGCCGGTGAATATCCAGGAAGCGGAGAGTGCGGTTTTTCAGATTAGGTAGTCGAAAAACCTAAGAAATTTAGGTGCTACATTTCAAGATAAA-3' |
| PK1 Deletion | 5'-AAAGCAAAAATGTGATCTTGCTTGTAAATACAATTTTGAGAGGTTAATAAATTACAAGTAGTGCTATTTTTGTATTTAGGTTAGCTATTTAGCTTTACGTTCCAGGATGCCTAGTGGCAGCCCCACAATATCCAGGAAGCCCTCTCTGCCTACATTTCAAGATAAA-3' |
| PK2+PK3 Deletion | 5'-GCGGTTTTTCAGATTAGGTAGTCGAAAAACCTAAGAAATTTACCTGCTACATTTCAAGATAAA-3' |
| Scrambled | 5'-GTAAGATGTTTCATGACCAACCCTAAATCTAAGTATATAAGTTGCACTTGAAAATGGTAATTCTTAATAAGCTGGATGAGTAACGAGTATTATTATCGAACGGAATTTTCCTGGTGAAACACTCTTTCATGACACGGAAATAAGGTGCGTCTTCTTTCAATATATCTCACGATGGCTTGAACAGACGGTTTCATAACTTTCTTAAATGCG-3' |
| PK2_Opp | 5'-AAAGCAAAAATGTGATCTTGCTTGTAAATACAATTTTCTCTCCTTAATAAATTACAAGTAGTGCTATTTTTGTATTTAGGTTAGCTATTTAGCTTTACGTTCCAGGATGCCTAGTGGCAGCCCCACAATATCCAGGAAGCCCTCTCTGCGGTTTTTCAGATTAGGTAGTCGAAAAACCTAAGAAATTTACCTGCTACATTTCAAGATAAA-3' |
| PK2_Comp | 5'-AAAGCAAAAATGTGATCTTGCTTGTAAATACAATTTTCTCTCCTTAATAAATTACAAGTAGTGCTATTTTTGTATTTAGGTTAGCTATTTAGCTTTACGTTCCAGGATGCCTAGTGGCAGCCCCACAATATCCAGGAAGCGGAGAGTGCGGTTTTTCAGATTAGGTAGTCGAAAAACCTAAGAAATTTACCTGCTACATTTCAAGATAAA-3' |
| IAPV | Sequence Upstream from sfGFP |
| WT | 5'-GAGCGGTTTCTGGAATACTATATGTAAGTATAGTGTTCTGGAGGCATCATTCTATGGTTACCCATCATTAGAGGAAATTTCCAATAAACTCTGGTGTAAGGCTTAGAGTGATGGTCGAGGTGCCCTATTTAGGGTGAGGAGCCTCGGTGGCAGCCCCACCAAATCCTCTATTGGATAGGAACAGCTGTACTGGGCAGTTACAGCAGTCGTATGGTAACACATGCGGCGTTCCGAAATACCATGCCTGGCGATTCACAACAAGAA-3' |
| PK1_K/O | 5'-GAGCGGTTTCTGGAATACTATATGTAAGTATAGTGTTCTGGAGGCATCATTCTATGGTTACCCATCATTAGAGGAAATTTCCAATAAACTCTGGTGTAAGGCTTAGAGTGATGGTCGAGGTGCCCTATTTAGGGTGAGGAGCCTCGGTGGCAGCCCCACCAAATCCTCTATTGGATAGGAACAGCTGTACTGGGCAGTTACAGCAGTCGTATGGTAACACATGCGGCGTTCCGAAATACCATGGGTGGCGATTCACAACAAGAA-3' |
| PK2_K/O | 5'-GAGCGGTTTCTGGAATACTATATGTAAGTATAGTGTTCTGGAGGCATCATTCTATGGTTACCCATCATTAGAGGAAATTTCCAATAAACTCTGGTGTAAGGCTTAGAGTGATGGTCGAGGTGCCCTATTTAGGGTGAGGAGCCTCGGTGGCAGCCCCACCAAATCCTCTTAACCATAGGAACAGCTGTACTGGGCAGTTACAGCAGTCGTATGGTAACACATGCGGCGTTCCGAAATACCATGCCTGGCGATTCACAACAAGAA-3' |
| PK3_K/O | 5'-GAGCGGTTTCTGGAATACTATATGTAAGTATAGTGTTCTGGAGGCATCATTCTATGGTTACCCATCATTAGAGGAAATTTCCAATAAACTCTGGTGTAAGGCTTAGAGTGATGGTCGAGGTGCCCTATTTAGGGTGAGGAGCCTCGGTGGCAGCCCCTGGAAATCCTCTATTGGATAGGAACAGCTGTACTGGGCAGTTACAGCAGTCGTATGGTAACACATGCGGCGTTCCGAAATACCATGCCTGGCGATTCACAACAAGAA-3' |
| PK1+PK3_K/O | 5'-GAGCGGTTTCTGGAATACTATATGTAAGTATAGTGTTCTGGAGGCATCATTCTATGGTTACCCATCATTAGAGGAAATTTCCAATAAACTCTGGTGTAAGGCTTAGAGTGATGGTCGAGGTGCCCTATTTAGGGTGAGGAGCCTCGGTGGCAGCCCCTGGAAATCCTCTATTGGATAGGAACAGCTGTACTGGGCAGTTACAGCAGTCGTATGGTAACACATGCGGCGTTCCGAAATACCATGGGTGGCGATTCACAACAAGAA-3' |
| PK1+PK2+PK3_K/O | 5'-GAGCGGTTTCTGGAATACTATATGTAAGTATAGTGTTCTGGAGGCATCATTCTATGGTTACCCATCATTAGAGGAAATTTCCAATAAACTCTGGTGTAAGGCTTAGAGTGATGGTCGAGGTGCCCTATTTAGGGTGAGGAGCCTCGGTGGCAGCCCCTGGAAATCCTCTTAACCATAGGAACAGCTGTACTGGGCAGTTACAGCAGTCGTATGGTAACACATGCGGCGTTCCGAAATACCATGGGTGGCGATTCACAACAAGAA-3' |
| Scrambled | 5'-CCCGTATGGGGATCGGACCGTGTTGCGCGCGATTGGATATACAAGCATAATTCTAAAAGGGACCTTGTCTGGGTTATCACTCCGATCTTGCGTTAACCATTATGTTATAACGCAGACTATTGAGCCGCGAGACAAAGCCCCTTTGATTTTAAGTGACATCACTAGGTCAAGCAGCAAGGTCTGGCAGACTAGGTATTAAGTAAAGTGTGCATACGGTGAGTGTTGAGGCGCCGCCAGTCAGCGGGATAAAAGATCTTAGCTTAT-3' |
| PSIV | Sequence Upstream from sfGFP |
| WT | 5'-AAGCTGACTATGTGATCTTATTAAAATTAGGTTAAATTTCGAGGTTAAAAATAGTTTTAATATTGCTATAGTCTTAGAGGTCTTGTATATTTATACTTACCACACAAGATGGACCGGAGCAGCCCTCCAATATCTAGTGTACCCTCGTGCTCGCTCAAACATTAAGTGGTGTTGTGCGAAAAGAATCTCACTTCAAGAAAAAGAATTTACC-3' |
| PK1_K/O | AAGCTGACTATGTGATCTTATTAAAATTAGGTTAAATTTCGAGGTTAAAAATAGTTTTAATATTGCTATAGTCTTAGAGGTCTTGTATATTTATACTTACCACACAAGATGGACCGGAGCAGCCCTCCAATATCTAGTGTACCCTCGTGCTCGCTCAAACATTTTCACGTGTTGTGCGAAAAGAATCTCACTTCAAGAAAAAGAATTTACC-3' |
| PK2_K/O | 5'-AAGCTGACTATGTGATCTTATTAAAATTAGGTTAAATTTCGAGGTTAAAAATAGTTTTAATATTGCTATAGTCTTAGAGGTCTTGTATATTTATACTTACCACACAAGATGGACCGGAGCAGCCCTCCAATATCTAGTGTACGGAGCTGCTCGCTCAAACATTAAGTGGTGTTGTGCGAAAAGAATCTCACTTCAAGAAAAAGAATTTACC-3' |
| PK2_Opp | 5'-AAGCTGACTATGTGATCTTATTAAAATTAGGTTAAATTTGCTCCTTAAAAATAGTTTTAATATTGCTATAGTCTTAGAGGTCTTGTATATTTATACTTACCACACAAGATGGACCGGAGCAGCCCTCCAATATCTAGTGTACCCTCGTGCTCGCTCAAACATTAAGTGGTGTTGTGCGAAAAGAATCTCACTTCAAGAAAAAGAATTTACC-3' |
| PK2_Comp | 5'-AAGCTGACTATGTGATCTTATTAAAATTAGGTTAAATTTGCTCCTTAAAAATAGTTTTAATATTGCTATAGTCTTAGAGGTCTTGTATATTTATACTTACCACACAAGATGGACCGGAGCAGCCCTCCAATATCTAGTGTACGGAGCTGCTCGCTCAAACATTAAGTGGTGTTGTGCGAAAAGAATCTCACTTCAAGAAAAAGAATTTACC-3' |
| PK3_K/O | 5'-AAGCTGACTATGTGATCTTATTAAAATTAGGTTAAATTTCGAGGTTAAAAATAGTTTTAATATTGCTATAGTCTTAGAGGTCTTGTATATTTATACTTACCACACAAGATGGACCGGAGCAGCCGAGGAATATCTAGTGTACCCTCGTGCTCGCTCAAACATTAAGTGGTGTTGTGCGAAAAGAATCTCACTTCAAGAAAAAGAATTTACC-3' |
| PK1+PK3_K/O | 5'-AAGCTGACTATGTGATCTTATTAAAATTAGGTTAAATTTCGAGGTTAAAAATAGTTTTAATATTGCTATAGTCTTAGAGGTCTTGTATATTTATACTTACCACACAAGATGGACCGGAGCAGCCGAGGAATATCTAGTGTACCCTCGTGCTCGCTCAAACATTTTCACGTGTTGTGCGAAAAGAATCTCACTTCAAGAAAAAGAATTTACC-3' |
| PK1+PK2+PK3_K/O | 5'-AAGCTGACTATGTGATCTTATTAAAATTAGGTTAAATTTCGAGGTTAAAAATAGTTTTAATATTGCTATAGTCTTAGAGGTCTTGTATATTTATACTTACCACACAAGATGGACCGGAGCAGCCGAGGAATATCTAGTGTACGGAGCTGCTCGCTCAAACATTTTCACGTGTTGTGCGAAAAGAATCTCACTTCAAGAAAAAGAATTTACC-3' |
| SDS-like_K/O | 5'-AAGCTGACTATGTGATCTTATTAAAATTAGGTTAAATTTCGAGGTTAAAAATAGTTTTAATATTGCTATAGTCTTAGAGGTCTTGTATATTTATACTTACCACACAAGATGGACCGGAGCAGCCCTCCAATATCTAGTGTACCCTCGTGCTCGCTCAAACATTAAGTGGTGTTGTGCGAAAAGAATCTCACTTCAACTGGTCTTGACGGCC-3' |
| RBS | Sequence Upstream from sfGFP |
| Strong (BBa_B0034) | 5'-AAAGAGGAGAAATACTAG-3' |
| Medium (BBa_B0032) | 5'-TCACACAGGAAAGTACTAG-3' |
| Weak (BBa_B0033) | 5'-TCACACAGGACTACTAG-3' |
| Dead (BBa- B0034 Inv) | 5'-TTTCTCCTCTTTACTAG-3' |

*All PSIV bicistronic constructs are identical to the monocistronic constructs except they have the following sequence (coding for T7-RBS-mRFP) upstream of the IGR IRES: 5’-TAATACGACTCACTATAGGGAGATACTAGAGTCACACAGGACTACTAGATGGCTTCCTCCGAAGACGTTATCAAAGAGTTCATGCGTTTCAAAGTTCGTATGGAAGGTTCCGTTAACGGTCACGAGTTCGAAATCGAAGGTGAAGGTGAAGGTCGTCCGTACGAAGGTACCCAGACCGCTAAACTGAAAGTTACCAAAGGTGGTCCGCTGCCGTTCGCTTGGGACATCCTGTCCCCGCAGTTCCAGTACGGTTCCAAAGCTTACGTTAAACACCCGGCTGACATCCCGGACTACCTGAAACTGTCCTTCCCGGAAGGTTTCAAATGGGAACGTGTTATGAACTTCGAAGACGGTGGTGTTGTTACCGTTACCCAGGACTCCTCCCTGCAAGACGGTGAGTTCATCTACAAAGTTAAACTGCGTGGTACCAACTTCCCGTCCGACGGTCCGGTTATGCAGAAAAAAACCATGGGTTGGGAAGCTTCCACCGAACGTATGTACCCGGAAGACGGTGCTCTGAAAGGTGAAATCAAAATGCGTCTGAAACTGAAAGACGGTGGTCACTACGACGCTGAAGTTAAAACCACCTACATGGCTAAAAAACCGGTTCAGCTGCCGGGTGCTTACAAAACCGACATCAAACTGGACATCACCTCCCACAACGAAGACTACACCATCGTTGAACAGTACGAACGTGCTGAAGGTCGTCACTCCACCGGTGCTTAATAACGCTGATAGTGCTAGTGTAGATCGCTACTAGAG-3’

**Table S2.** DNA sequences for control RNAs used in filter binding.

| **Control RNAs** | **Sequences used in filter binding** |
| --- | --- |
| *rpsO* | 5'-GGATCCTAATACGACTCACTATAGGTACGAGTAGAATACTGCCGCTTAACGTCGCGTAAATTGTTTAACACTTTGCGTAACGTACACTGGGATCGCTGAATTAGAGATCGGCGTCCTTTCATTCTATATACTTTGGAGTTTTAAAATGTCTCAAGTACTGAAGCAACAGCTAAAATCGTTTCTGAGTTTGGTCGTGACGCAAACGACACC-3' |
| *sodB* | 5'-GGATCCTAATACGACTCACTATAGGATACGCACAATAAGGCTATTGTACGTATGCAAATTAATAATAAAGGAGAGTAGCAATGTCATTCGAATTACCTGCACTACCATATGCTAAAGATGCTCTGGCACCGCACATTTCTGCGG-3' |
| tRNA^Phe^ | 5'-GCGCGGAUGCUCAGUCGGUAGAGCAGGGGAUUGAAAAUCCCCGUGUCCUUGGUUCGAUUCCGAGUCCGCGCACCA-3' |

**Table S3.** Equilibrium dissociation constants and percent of RNA bound for CrPV IGR IRES variants and the 40S (HeLa) ribosomal subunit. Mean values of three replicates are reported; error indicates one standard deviation.

| CrPV Construct | K_D_ (nM) | Percent Bound (%) |
| --- | --- | --- |
| Wild type | 14 ± 8 | 97 ± 7 |
| PK1_K/O | 4 ± 2 | 34 ± 2 |
| PK1+PK3_K/O | No Binding | No Binding |

**Table S4.** Equilibrium dissociation constants and percent of RNA bound for control RNAs and the 30S ribosome. Mean values of three replicates are reported; error indicates one standard deviation.

| Construct | K_D_ (nM) | Percent Bound (%) |
| --- | --- | --- |
| rpsO | 10 ± 5 | 39 ± 2 |
| sodB | 13 ± 2 | 41 ± 3 |
| tRNA^Phe^ | 35 ± 10 | 10 ± 1 |

**Table S5.** Equilibrium dissociation constants and percent of RNA bound for control RNAs and the 70S ribosome. Mean values of three replicates are reported; error indicates one standard deviation.

| Construct | K_D_ (nM) | Percent Bound (%) |
| --- | --- | --- |
| rpsO | 94 ± 14 | 44 ± 10 |
| sodB | 47 ± 13 | 56 ± 8 |
| tRNA^Phe^ | No Binding | No Binding |
